# Supplementary material for: Anti-malarial contact dependent blocking of transmission of Plasmodium vivax by Anopheles darlingi mosquito vector
Source: PLoS Pathog. 2026 Jul 2;22(7):e1013531. doi: 10.1371/journal.ppat.1013531 (PMC13327285; doi:10.1371/journal.ppat.1013531)
Supplement: S2 Table — (DOCX) [file ppat.1013531.s002.docx]

**S2 Table.** Characteristics of *Plasmodium vivax* obtained from patients and used in *ex vivo* assay with Atovaquone (ATQ).

| ID | Sex | Parasites/ µL | Trophozoites  0 hour (%) | Schizontes  mature (%) | Incubation Time | IC_50_µM |
| --- | --- | --- | --- | --- | --- | --- |
|  |  |  |  |  |  | **ATQ** |
| C4 | F | 6510 | 95 | 40 | 48 | 0,00032 |
| C5 | M | 5130 | 90 | 55 | 48 | 0,00010 |
| C8 | F | 5636 | 96 | 45 | 48 | 0,00012 |
| C10  C11 | M  M | 4650  3930 | 91  89 | 55  60 | 49  51 | 0,000019  0,000041 |

**Methods**

After approval by the Research Ethics Committee (CEP) CAAE: 56853922.2.0000.5300 and number 5.832.189), patients positive to *P. vivax* who voluntarily sought care at the Center for Research in Tropical Medicine (CEPEM) in Porto Velho, RO, were invited to participate in the study through and informed consent from. The *ex vivo* assays were conducted with five isolates, using convenience samples from patietens with confirmed parasitemia above 2,000 parasites/mm³ by thick blood smear. The *ex vivo* chemosensitivity plates were prepared with the antimalarial ATQ, tested in serial 1:4 dilutions with final concentration of 0.078 µM. To achieve this, parasites were separated from leukocytes using a CF11 cellulose column [1]​. Subsequently, parasitemia was adjusted to a hematocrit level of 2% using IMDM (Iscove’s Modified Dulbecco’s Medium) supplemented with 20% human AB serum for *P*. *vivax*. At the end of the process, the culture was exposed to a gas mixture and incubated at 37 ºC. The control (DMSO with same concentration used to treatment group) was monitored through thick blood examination to verify parasite maturation at the schizont stage. The assay was halted when parasitemia reached ≥ 40% schizonts in 200 parasites forms.

The assay results were determined by optical microscopy (100x) after 24-48 hours of incubation. For thick blood smear preparation, 2 µL of culture sample were initially used, followed by Giemsa staining [2,3]​.

​**References**

1. Sriprawat K, Kaewpongsri S, Suwanarusk R, Leimanis ML, Lek-Uthai U, Phyo AP, et al. Effective and cheap removal of leukocytes and platelets from *Plasmodium vivax* infected blood. Malar J. 2009; 8(1):115. doi: 10.1186/1475-2875-8-115

2. Aguiar ACC, Pereira DB, Amaral NS, De Marco L, Krettli AU. *Plasmodium vivax* and *Plasmodium falciparum ex vivo* susceptibility to anti-malarials and gene characterization in Rondônia, West Amazon, Brazil. Malar J. 2014;13(1):73. doi: 10.1186/1475-2875-13-73

3. Marfurt J, Chalfein F, Prayoga P, Wabiser F, Kenangalem E, Piera KA, et al. *Ex Vivo* Drug Susceptibility of Ferroquine against Chloroquine-Resistant Isolates of *Plasmodium falciparum* and *P. vivax*. Antimicrob Agents Chemother. 2011;55(9):4461–4. doi: 10.1128/AAC.01375-10
