# Supplementary material for: Anti-malarial contact dependent blocking of transmission of Plasmodium vivax by Anopheles darlingi mosquito vector
Source: PLoS Pathog. 2026 Jul 2;22(7):e1013531. doi: 10.1371/journal.ppat.1013531 (PMC13327285; doi:10.1371/journal.ppat.1013531)
Supplement: S3 Table — (DOCX) [file ppat.1013531.s003.docx]

**S3 Table.** Characteristics of *P. vivax* obtained from patients and used in ookinete inhibition assay with Atovaquone (ATQ).

| **ID** | **Gametocytes (µL)** | **Imature stage**  **(µL)** | **Madure**  **stage**  **(µL)** | **Total of**  **ookinetes**  **(µL)** | **Ookinete inhibition 10 μM (%)** |
| --- | --- | --- | --- | --- | --- |
|  |  |  |  |  | **ATQ** |
| ID01 | 300 | 935 | 893 | 1828 | 93.8 ± 2.0 |
| ID02 | 690 | 68 | 1245 | 1313 | 91.5 ± 2.8 |
| ID05 | 1230 | 200 | 875 | 1075 | 99.8 ± 0.2 |
| ID08 | 300 | 857 | 68 | 925 | 99.8 ± 0.3 |
| ID11 | 1200 | 518 | 4373 | 4892 | 96.9 ± 5.2 |
| ID16 | 1200 | 518 | 632 | 1150 | 99.4 ± 0.25 |
| ID20 | 120 | 1096 | 798 | 1895 | 100 ± 0.01 |
| A1-23 | 570 | 3797 | 2510 | 6307 | 99.8 ± 0.05 |
| A2-23 | 210 | 830 | 1077 | 1907 | 87.0 ± 4.40 |
| A7-24 | 411 | 930 | 297 | 1226 | 84.5 ± 0.82 |
| Mean | 623 | 974 | 1276 | 2251 | 95.2 ± 1.6 |

Mean inhibition of *P. vivax* ookinetes per microliter of blood using microscopy counting from five independent samples. ATQ was solubilized in DMSO (Dimethyl sulfoxide) not exceeding a concentration of 0.5% and DMSO was used as control.

**Methods**

After collecting the infected samples, a Giemsa staining slides were prepared to be quantified gametocytemia of each sample. The number of parasites must be equal to or greater than 10 gametocytes per 200 leukocytes. Following this confirmation, the process of washing the red blood cells with incomplete RPMI-1640 medium is initiated through centrifuge at 678 xg for 10 minutes to remove leukocytes. After two washes, gametocyte separation is carried out using a 55% Histodenz purification gradient diluted in 1x PBS with a final pH of 7.2 (Phosphate Buffered Saline) at a 1:3 ratio relative to the sample volume. This gradient is prepared from a stock solution [27.6% (w/v) Histodenz in 5.0 mM tris-HCl, 3.0 mM KCl, and 0.3 mM EDTA (pH 7.20)] [1]. After this step, the sample is centrifuge once at 1500 x g for 20 minutes without braking. At the end of the centrifugation process, the pellet remaining at the interface is collected and washed twice at 800 x g for 3 minutes. Subsequently, the hematocrit is adjusting to 5% using ookinete medium supplemented with 20% human serum and 2.5% de Albumax. The gametocytes are then added to the wells of 96-well plates containing the ATQ, which have been pre-diluted at concentrations 10 µM. DMSO (0.5%) is used as the negative inhibition control [2]. Finally, the parasites are incubated for 24 hours at temperature of 21-24 ºC. After this period, compound activity is assessed through microscopy counting across 100 field to estimate parasitemia per µL in each sample.

**References**

1. Balestra AC, Koussis K, Klages N, Howell SA, Flynn HR, Bantscheff M, et al. Ca2+ signals critical for egress and gametogenesis in malaria parasites depend on a multipass membrane protein that interacts with PKG. Sci Adv. 2021;7(13):eabe5396. do: 10.1126/sciadv.abe5396

2. Delves MJ, Miguel-Blanco C, Matthews H, Molina I, Ruecker A, Yahiya S, et al. A high throughput screen for next-generation leads targeting malaria parasite transmission. Nat Commun. 2018;9(1):3805. doi: 10.1038/s41467-018-05777-2
