# Supplementary material for: Anti-malarial contact dependent blocking of transmission of Plasmodium vivax by Anopheles darlingi mosquito vector
Source: PLoS Pathog. 2026 Jul 2;22(7):e1013531. doi: 10.1371/journal.ppat.1013531 (PMC13327285; doi:10.1371/journal.ppat.1013531)
Supplement: S4 Table — (DOCX) [file ppat.1013531.s004.docx]

**S4 Table.** Chemical properties from all compound’s testes in this study – atovaquone, primaquine, tafenoquine, chloroquine, mefloquine and nanchangmycin.

| Compound | Molecular weight (g/mol) | LogP | PSA (Â^2^) | Target | Group | Vehicle | Plasmodial action | Human *Plasmodium* target | Metabolism |
| --- | --- | --- | --- | --- | --- | --- | --- | --- | --- |
| ATQ | 367.1 | 5.34 | 54.4 | Cytochrome bc1^1,2,3^  inhibition of pyrimidine synthesis^4^ | Naphthoquinone | Acetone | Blood^4,5,6^, liver ^5,7^ and sporogony stages^5^ | *P. falciparum*^7,4,5,6^ | No need to be metabolized^8^ |
| PQ | 455.34 | 2.78 | 60.15 | Hemozoin^9,10^ | 8-aminoquinolines | Ethanol | Hypnozoite^11,12,14^, gametocyte^16,13,15^, schizont tissue^16^ and sporogonic^16^ | *P. vivax*^11,12^*, P. ovale*^11,12^ *e P. falciparum*^7^*^,^*^31^ | Metabolized in liver^9,10^ |
| TQ | 581.58 | 5.91 | 78.63 | Unknown^17^ | 8-aminoquinolines | Methanol | Hypnozoite^11,14^ and Gametocyte^15,16,17^. | *P. viva*^11,18^ and *P. falciparum*^18,19^ | Minimal hepatic metabolism^17,19^ |
| CQ | 319.88 | 4.81 | 28.16 | Hemozoin^16,20,22,23,24^ | 4-aminoquinolines | Ethanol | Blood schizont^16,25^ asexual and young Gametocyte^16,25^ | *P. vivax*^16^*, P. falciparum*^25^  and *P. malariae*^16^ | Metabolized in liver^26^ |
| MQ | 463.5 | 4.44 | 45.15 | Hemozoin^16,27,28^ | Arylamino alcohols^29^ | Ethanol | Blood schizont- asexual^11,16,27^ | *P. vivax, P. malariae* and *P. falciparum* | Metabolized in liver  Partially metabolized in liver^30^ |
| NCG | 867.1 | 5.8 | 191.7 | Unknown | Polyether ionophore^31^ | Ethanol | Exflagellation (male gametocyte) and oocyst^32^ | *P. vivax, P. falciparum* and *P. berghei*^32^ |  |

PSA: Polar Surface Area; LogP: Partition coefficient. ATQ: Atovaquone; CQ: Chloroquine; MQ: Mefloquine; NCG: Nanchangmycin; PQ: Primaquine; TQ: Tafenoquine.

**References**

1. Fry M, Pudney M. Site of action of the antimalarial hydroxynaphthoquinone, 2-[*trans*-4-(4’-chlorophenyl) cyclohexyl]-3- hydroxy-1,4-naphthoquinone (566C80). Biochem Pharmacol. 1992 Apr 1;43(7):1545–53. doi:10.1016/0006-2952(92)90213-3

2. Fleck SL, Pudney M, Sinden RE. The effect of atovaquone (566C80) on the maturation and viability of *Plasmodium falciparum* gametocytes *in vitro*. Trans R Soc Trop Med Hyg. 1996 May 1;90(3):309–12. https://doi.org/10.1016/S0035-9203(96)90266-7

3. Srivastava IK, Rottenberg H, Vaidya AB. Atovaquone, a Broad Spectrum Antiparasitic Drug, Collapses Mitochondrial Membrane Potential in a Malarial Parasite. J Biol Chem. 1997 Feb 14;272(7):3961–6. https://doi.org/10.1074/jbc.272.7.3961

4. Painter HJ, Morrisey JM, Mather MW, Vaidya AB. Specific role of mitochondrial electron transport in blood-stage *Plasmodium falciparum*. Nature. 2007 Mar;446(7131):88–91. https://doi.org/10.1038/nature05572

5. Delves M, Plouffe D, Scheurer C, Meister S, Wittlin S, Winzeler EA, et al. The Activities of Current Antimalarial Drugs on the Life Cycle Stages of *Plasmodium*: A Comparative Study with Human and Rodent Parasites. PLOS Med. 2012 Feb 21;9(2):e1001169. https://doi.org/10.1371/journal.pmed.1001169

6. Painter HJ, Morrisey JM, Vaidya AB. Mitochondrial Electron Transport Inhibition and Viability of Intraerythrocytic *Plasmodium falciparum*. Antimicrob Agents Chemother. 2010 Dec;54(12):5281–7. https://doi.org/10.1128/aac.00937-10

7. Berman JD, Nielsen R, Chulay JD, Dowler M, Kain KC, Kester KE, et al. Causal prophylactic efficacy of atovaquone-proguanil (MalaroneTM) in a human challenge model. Trans R Soc Trop Med Hyg. 2001 July 1;95(4):429–32. https://doi.org/10.1016/S0035-9203(01)90206-8

8. Balta VA, Stiffler D, Sayeed A, Tripathi AK, Elahi R, Mlambo G, et al. Clinically relevant atovaquone-resistant human malaria parasites fail to transmit by mosquito. Nat Commun. 2023 Oct 12;14(1):6415. https://doi.org/10.1038/s41467-023-42030-x

9. Pybus BS, Marcsisin SR, Jin X, Deye G, Sousa JC, Li Q, et al. The metabolism of primaquine to its active metabolite is dependent on CYP 2D6. Malar J. 2013 June 20;12(1):212. https://doi.org/10.1186/1475-2875-12-212

10. Camarda G, Jirawatcharadech P, Priestley RS, Saif A, March S, Wong MHL, et al. Antimalarial activity of primaquine operates via a two-step biochemical relay. Nat Commun. 2019 July 19;10(1):3226. https://doi.org/10.1038/s41467-019-11239-0

11. World Health Organization. Guidelines for the treatment of malaria. Third edition | WHO | Regional Office for Africa [Internet]. 2015. https://www.afro.who.int/publications/guidelines-treatment-malaria-third-edition

12. Nodiff EA, Chatterjee S, Musallam HA. 1 Antimalarial Activity of the 8-Aminoquinolines. In: Ellis GP, West GB, editors. Progress in Medicinal Chemistry [Internet]. Elsevier; 1991. p. 1–40. https://doi.org/10.1016/s0079-6468(08)70362-x

13. Shekalaghe S, Drakeley C, Gosling R, Ndaro A, Meegeren M van, Enevold A, et al. Primaquine Clears Submicroscopic *Plasmodium falciparum* Gametocytes that Persist after Treatment with Sulphadoxine-Pyrimethamine and Artesunate. PLOS ONE. 2007 Oct 10;2(10):e1023. https://doi.org/10.1371/journal.pone.0001023

14. Brito M, Rufatto R, Brito-Sousa JD, Murta F, Sampaio V, Balieiro P, et al. Operational effectiveness of tafenoquine and primaquine for the prevention of *Plasmodium vivax* recurrence in Brazil: a retrospective observational study. Lancet Infect Dis. 2024 June 1;24(6):629–38. https://doi.org/10.1016/S1473-3099(24)00074-4

15. Andrade AO, Santos NAC, Bastos AS, Pontual JDC, Araújo CS, Lima AS, et al. Optimization of *Plasmodium vivax* infection of colonized Amazonian *Anopheles darlingi*. Sci Rep. 2023 Oct 24;13(1):18207. https://doi.org/10.1038/s41598-023-44556-y

16. Vangapandu S, Jain M, Kaur K, Patil P, Patel SR, Jain R. Recent advances in antimalarial drug development. Med Res Rev. 2007;27(1):65–107. https://doi.org/10.1002/med.20062

17. Lu KY, Derbyshire ER. Tafenoquine: A Step toward Malaria Elimination. Biochemistry. 2020 Mar 3;59(8):911–20. https://doi.org/10.1021/acs.biochem.9b01105

18. McCarthy JS, Smith B, Reid M, Berman J, Marquart L, Dobbin C, et al. Blood Schizonticidal Activity and Safety of Tafenoquine When Administered as Chemoprophylaxis to Healthy, Nonimmune Participants Followed by Blood Stage *Plasmodium falciparum* Challenge: A Randomized, Double-blind, Placebo-controlled Phase 1b Study. Clin Infect Dis. 2019 July 18;69(3):480–6. https://doi.org/10.1093/cid/ciy939

19. National Institute of Diabetes and Digestive and Kidney Diseases. Tafenoquine. In: LiverTox: Clinical and Research Information on Drug-Induced Liver Injury [Internet]. Bethesda (MD): National Institute of Diabetes and Digestive and Kidney Diseases; 2012. http://www.ncbi.nlm.nih.gov/books/NBK548428/

20. Olafson KN, Ketchum MA, Rimer JD, Vekilov PG. Mechanisms of hematin crystallization and inhibition by the antimalarial drug chloroquine. Proc Natl Acad Sci. 2015 Apr 21;112(16):4946–51. https://doi.org/10.1073/pnas.1501023112

21. Sullivan DJ. Quinolines block every step of malaria heme crystal growth. Proc Natl Acad Sci. 2017 July 18;114(29):7483–5. https://doi.org/10.1073/pnas.1708153114

22. de Dios AC, Tycko R, Ursos LMB, Roepe PD. NMR Studies of Chloroquine−Ferriprotoporphyrin IX Complex. J Phys Chem A. 2003 July 1;107(30):5821–5. https://doi.org/10.1021/jp0342982

23. Egan TJ, Ross DC, Adams PA. Quinoline anti-malarial drugs inhibit spontaneous formation of β-haematin (malaria pigment). FEBS Lett. 1994;352(1):54–7. https://doi.org/10.1016/0014-5793(94)00921-X

24. Padmanaban G, Rangarajan PN. Heme Metabolism of *Plasmodium* Is a Major Antimalarial Target. Biochem Biophys Res Commun. 2000 Feb 24;268(3):665–8. https://doi.org/10.1006/bbrc.1999.1892

25. Smalley ME, Sinden RE. *Plasmodium* falciparum gametocytes: their longevity and infectivity. Parasitology. 1977 Feb;74(1):1–8. https://doi.org/10.1017/s0031182000047478

26. Rendic S, Guengerich FP. Metabolism and Interactions of Chloroquine and Hydroxychloroquine with Human Cytochrome P450 Enzymes and Drug Transporters. Curr Drug Metab. 21(14):1127–35. https://doi.org/10.2174/1389200221999201208211537

27. Palmer KJ, Holliday SM, Brogden RN. Mefloquine. Drugs. 1993 Mar 1;45(3):430–75. https://doi.org/10.2165/00003495-199345030-00009

28. Dorn A, Vippagunta SR, Matile H, Jaquet C, Vennerstrom JL, Ridley RG. An Assessment of Drug-Haematin Binding as a Mechanism for Inhibition of Haematin Polymerisation by Quinoline Antimalarials. Biochem Pharmacol. 1998 Mar 15;55(6):727–36. https://doi.org/10.1016/s0006-2952(97)00510-8

29. Rathod GK, Jain M, Sharma KK, Das S, Basak A, Jain R. New structural classes of antimalarials. Eur J Med Chem. 2022 Nov 15;242:114653. https://doi.org/10.1016/j.ejmech.2022.114653

30. BRASIL. Ministério da Saúde. Farmanguinhos Artesunato+mefloquina [Internet]. 2021. https://www.far.fiocruz.br/wp-content/uploads/2021/04/Artesunato-mefloquina_Bula_Profissional.pdf

31. Wollesen M, Mikkelsen K, Tvilum MS, Vestergaard M, Wang M, Meyer RL, et al. Polyether Ionophore Antibiotics Target Drug-Resistant Clinical Isolates, Persister Cells, and Biofilms. Microbiol Spectr. 2023 June 8;11(4):e00625-23. https://doi.org/10.1128/spectrum.00625-23

32. Calit J, Araújo JE, Deng B, Miura K, Gaitán XA, Araújo M da S, et al. Novel Transmission-Blocking Antimalarials Identified by High-Throughput Screening of *Plasmodium berghei* Ookluc. Antimicrob Agents Chemother. 2023 Mar;67(4):e01465-22. https://doi.org/10.1128/aac.01465-22
