# Supplementary figures and images for: Anti-malarial contact dependent blocking of transmission of Plasmodium vivax by Anopheles darlingi mosquito vector
Source: PLoS Pathog. 2026 Jul 2;22(7):e1013531. doi: 10.1371/journal.ppat.1013531 (PMC13327285; doi:10.1371/journal.ppat.1013531)

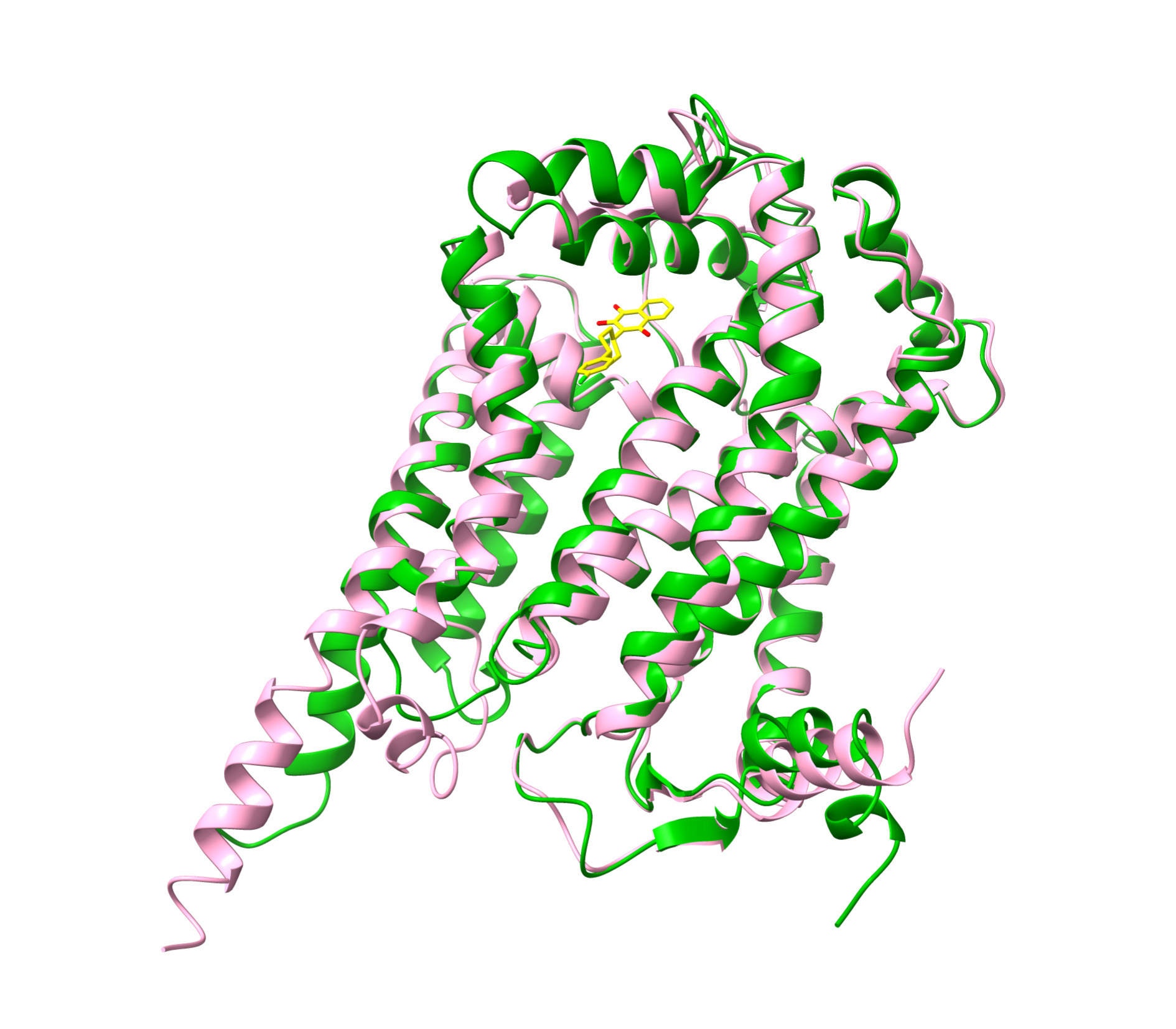

Supplement: S1 Fig — Structural superposition of Plasmodium vivax cytochrome b (light pink, AlphaFold model AF-O63696-F1-v4) and the C-chain of Saccharomyces cerevisiae cytochrome bc1 complex (green, PDB ID: 4pd4). Atovaquone (ATQ) is shown in yellow sticks as positioned in the crystallographic structure of Saccharomyces cerevisiae. The Root Mean Square Deviation (RMSD) between the two models was 0.824 Å, indicating high structural similarity. Structural visualization was performed in ChimeraX. (TIFF) [file ppat.1013531.s005.tiff]

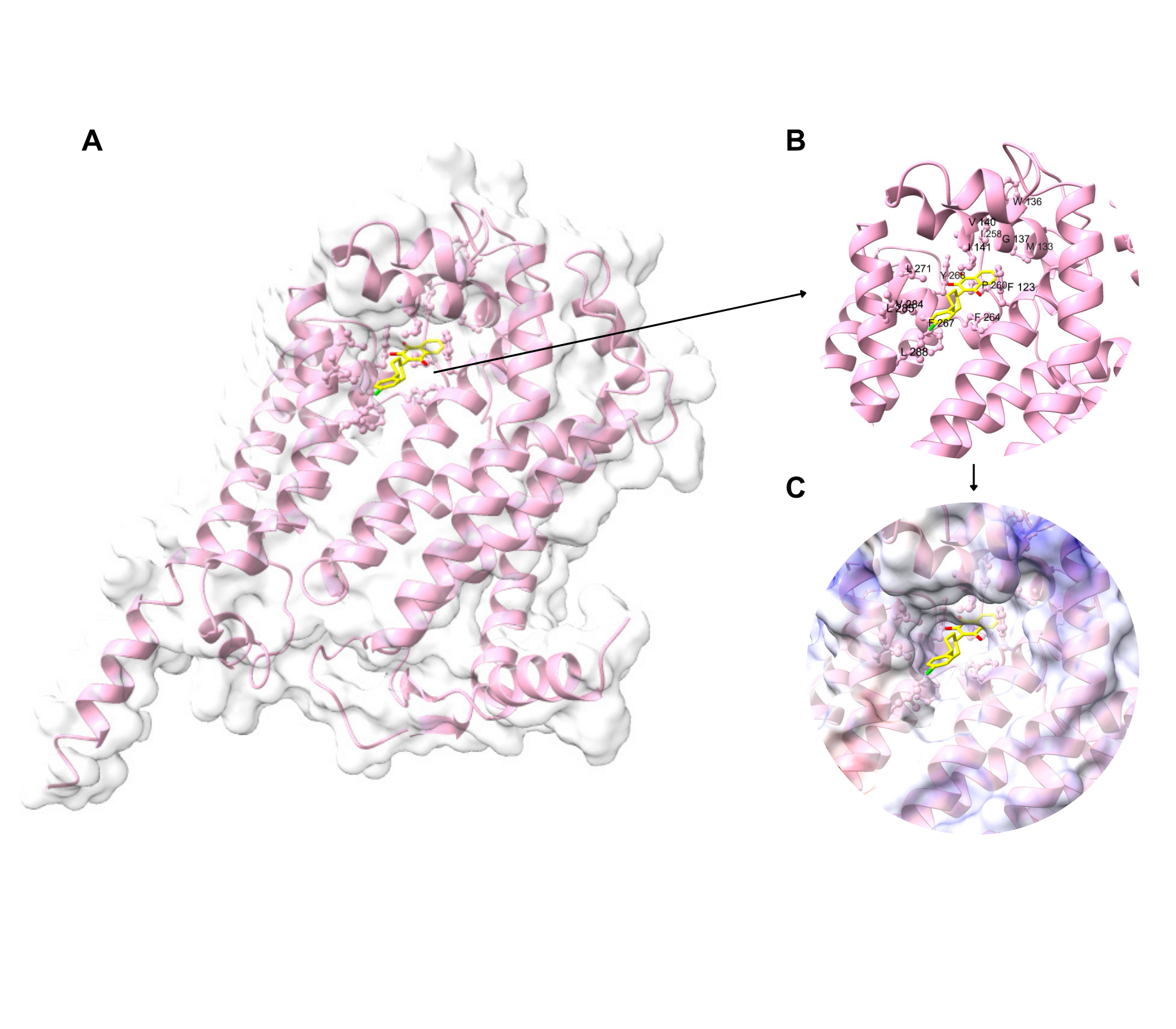

Supplement: S2 Fig — A) Tertiary structure of the cytochrome b (AlphaFold ID: AF-O63696-F1-v4) shown as a light pink ribbon. A transparent surface highlights the overall shape of the protein. ATQ is represented in yellow sticks. B) Close-up of ATQ-binding residues shown in ball-and-stick representation with residue names labeled (Phe123, Met133, Trp136, Gly137, Val140, Ile258, Leu285, Leu288, Pro260, Phe264, Tyr268, Leu271, Ile141, Phe267, and Val284). C) Electrostatic surface potential of the ATQ binding pocket. Red indicates negatively charged regions, blue indicates positively charged regions, and white indicates neutral areas. Structural visualization was performed in ChimeraX. (TIFF) [file ppat.1013531.s006.tiff]

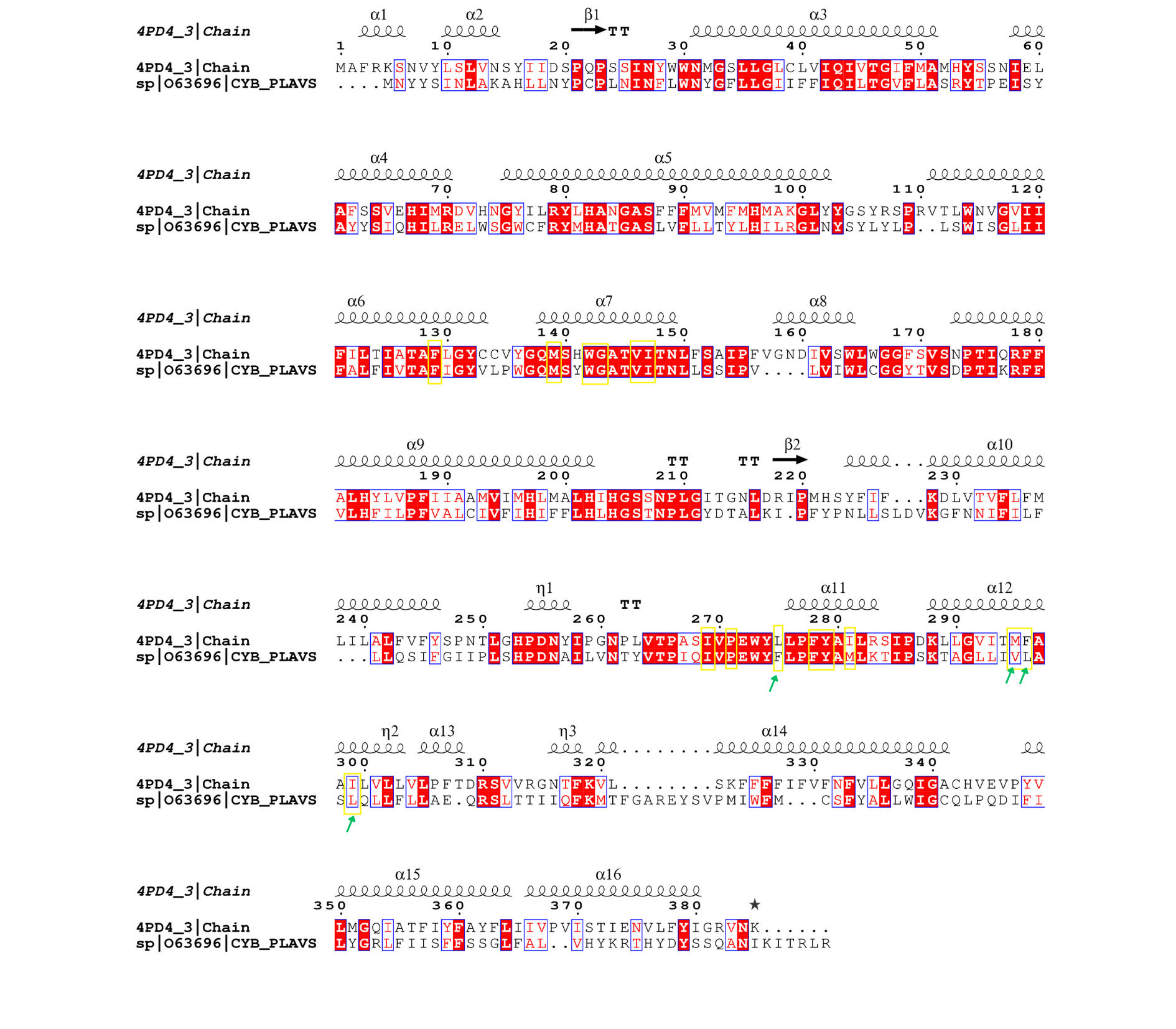

Supplement: S3 Fig — Sequence alignment of Plasmodium vivax cytb proteins (AF-O63696) and Saccharomyces cerevisiae Cytb C chain (PDB ID: 4pd4), performed using Clustalw. Residues highlighted in yellow correspond to those involved in atovaquone (ATQ) binding. Green arrows indicate residues that changed between proteins. Image made in ESPript 3.0. (TIFF) [file ppat.1013531.s007.tiff]
