# Supplementary material for: Anti-malarial contact dependent blocking of transmission of Plasmodium vivax by Anopheles darlingi mosquito vector
Source: PLoS Pathog. 2026 Jul 2;22(7):e1013531. doi: 10.1371/journal.ppat.1013531 (PMC13327285; doi:10.1371/journal.ppat.1013531)
Supplement: S1 File — (DOCX) [file ppat.1013531.s008.docx]

**S1 Method**

For a more robust outcome of the results, in addition to the *in vitro* and *ex vivo* experiments, an *in silico* analysis of the interaction of atovaquone (ATQ) with the molecular target cytb was performed. Thus, a preliminary structural and sequence comparison was performed between *P. vivax* and *Saccharomyces cerevisiae* cytb, in which the latter contains ATQ in the binding site.

Thus, for the structural analysis, the predicted structure of cytochrome b from *P. vivax*, strain El Salvador (AlphaFold ID: AF-O63696-F1-v4), obtained from the AlphaFold Protein Structure Database (<https://alphafold.ebi.ac.uk/>) and cytbc1 complex from *S. cerevisiae* cytbc1 complex S288C (PDB ID: 4pd4), more specifically the C chain, available in the RCSB Protein Data Bank (http://www.rcsb.org/) in which the inhibitor ATQ was already bound to the protein, were used.

The alignment of these sequences of the C chain of *S. cerevisiae* cytb (4pd4) and *P. vivax* cytb (AF-O63696) was performed in Clustal Omega Multiple Sequence Alignment (MSA) (https://www.ebi.ac.uk/jdispatcher/msa/clustalo).

The superposition of the protein structures, electrostatic surface potential and residues that interact with atovaquone were performed in the ChimeraX software version 1.8 (https://www.cgl.ucsf.edu/chimerax/). A Root Mean Square Deviation (RMSD) value of up to 1.2 Â was used for the structural superposition of the protein [1].

Regarding the preparation of the proteins, the structure of cytbc1 (PDB ID: 4pd4) with a resolution of 3.04 Â bound to atovaquone was used and using the PyMOL software (version 3.0.2) (http://www.pymol.org/), the ligand was removed from the PDB file and saved in a new file. After this procedure, the document with the isolated protein (cytbc1) and the ligand (ATQ) were subjected to molecular docking with the online software Dockthor (version 2.0) (https://dockthor.lncc.br/v2/) with grid dimensions of 20 x 20 x 20 Â and coordinates of X = 0.90, Y = 5.87 and Z = 12.05. To validate the docking performed, the ligand was subjected to redocking.

Using the coordinates used to anchor the ATQ in cytbc1 of *S. cerevisiae*, it was possible to anchor the ATQ in the predicted structure of cytb of *P. vivax* (AlphaFold ID: AF-O63696-F1-v4).

The protonation state was assigned at pH 6.8 considering a previous study that considered the ionized form of atovaquone [2].

**References**

1. Kufareva I, Abagyan R. Methods of protein structure comparison. Methods Mol Biol Clifton NJ. 2015;857:231–57. https://doi.org/10.1007/978-1-61779-588-6_10

2. Birth D, Kao WC, Hunte C. Structural analysis of atovaquone-inhibited cytochrome bc1 complex reveals the molecular basis of antimalarial drug action. Nat Commun. 2014 June 4;5(1):4029. https://doi.org/10.1038/ncomms5029
