# Supplementary material for: Anti-malarial contact dependent blocking of transmission of Plasmodium vivax by Anopheles darlingi mosquito vector
Source: PLoS Pathog. 2026 Jul 2;22(7):e1013531. doi: 10.1371/journal.ppat.1013531 (PMC13327285; doi:10.1371/journal.ppat.1013531)
Supplement: Attachment — Submitted filename: Comments from the Editor.docx [file ppat.1013531.s010.docx]

Dear Editor-in-Chief and Referees:

Thank you for your kind letter of “Decision on your submission PATHOGENS-D-25-02262” on the Nov 4^th^, 2025. The title of the manuscript is: “**Anti-malarial contact dependent blocking of transmission of *Plasmodium vivax* by *Anopheles darlingi* mosquito vector**” by Jéssica E. A. Kassupá, Alice O. Andrade, Alessandra S. Bastos, Gabriel L. L. Moura, Marina L. Rocha, Leandro do Nascimento Martinez, Elisandra Kathellen da Silva Afonso, Wallyson Costa, Daniel Y. Bargieri, Carolina Bioni Garcia Teles, Jansen F. Medeiros, Nirbhay Kumar, Ana Carolina Ramos Guimarães, Douglas G. Paton, Flaminia Catteruccia, and Maisa S. Araujo

We are very grateful to your and the reviewers’ comments and thoughtful suggestions. Based on these comments and suggestions, we have made careful modifications to the original manuscript and carefully proof-read the manuscript to minimize errors. We believe that the manuscript has been greatly improved and hope it has reached your journal’s standard.

Once again, we acknowledge your comments very much, which are valuable in improving the quality of our manuscript.

Sincerely yours

Maisa S. Araújo

Here are our responses to the reviewers’ comments and list of corrections and all revisions made to manuscript was marked up using the “Track Changes” as suggested.

**Journal Requirements:**

If the reviewer comments include a recommendation to cite specific previously published works, please review and evaluate these publications to determine whether they are relevant and should be cited. There is no requirement to cite these works unless the editor has indicated otherwise.

1) We do not publish any copyright or trademark symbols that usually accompany proprietary names, eg ©,  ®, or TM  (e.g. next to drug or reagent names). Therefore please remove all instances of trademark/copyright symbols throughout the text, including:
- ® on page: 22.

Answer’s author: We deleted it (Line 770).

2) Some material included in your submission may be copyrighted. According to PLOSu2019s copyright policy, authors who use figures or other material (e.g., graphics, clipart, maps) from another author or copyright holder must demonstrate or obtain permission to publish this material under the Creative Commons Attribution 4.0 International (CC BY 4.0) License used by PLOS journals. Please closely review the details of PLOSu2019s copyright requirements here: PLOS Licenses and Copyright. If you need to request permissions from a copyright holder, you may use PLOS's Copyright Content Permission form.
Please respond directly to this email and provide any known details concerning your material's license terms and permissions required for reuse, even if you have not yet obtained copyright permissions or are unsure of your material's copyright compatibility. Once you have responded and addressed all other outstanding technical requirements, you may resubmit your manuscript within Editorial Manager. 
Potential Copyright Issues:
i) Figures 1A, 1C, 2A, 2B, 3, 4, and 5. Please confirm whether you drew the images / clip-art within the figure panels by hand. If you did not draw the images, please provide (a) a link to the source of the images or icons and their license / terms of use; or (b) written permission from the copyright holder to publish the images or icons under our CC BY 4.0 license. Alternatively, you may replace the images with open source alternatives. See these open source resources you may use to replace images / clip-art:
- https://commons.wikimedia.org
- https://openclipart.org/.

Answer’s author: All the figures, graphics and clipart were manually designed using Canva.

3) Please amend your detailed Financial Disclosure statement. This is published with the article. It must therefore be completed in full sentences and contain the exact wording you wish to be published.
1) State the initials, alongside each funding source, of each author to receive each grant. For example: "This work was supported by the National Institutes of Health (####### to AM; ###### to CJ) and the National Science Foundation (###### to AM)."
2) State what role the funders took in the study. If the funders had no role in your study, please state: "The funders had no role in study design, data collection and analysis, decision to publish, or preparation of the manuscript."
3) If any authors received a salary from any of your funders, please state which authors and which funders..
If you did not receive any funding for this study, please simply state: u201cThe authors received no specific funding for this work.u201d
4)  Please ensure that the funders and grant numbers match between the Financial Disclosure field and the Funding Information tab in your submission form. Note that the funders must be provided in the same order in both places as well.

Answer’s author: Please, use this sentence: “This research was funded by the Conselho Nacional de Desenvolvimento Científico e Tecnológico CNPq/MCTI/FNDCT Nº 19/2024 – Centros Avançados em Áreas Estratégicas para o Desenvolvimento Sustentável da Região Amazônica - Pro- Amazônia [N° 444882/2024-3] (J.F.M and M.S.A), Programa de Excelência em Pesquisa – PROEP (PRES-028-FIO-24-2-11 to J.F.M) and NIH grant R01-AI183533 to NK. The facilities (Rede de Plataformas Tecnológicas Fiocruz: RPT) used in this study were funded by Fiocruz (M.S.A). J.M.F. is is CNPq productivity fellows (process number: 304830/2022-4). The funders had no role in study design, data collection and analysis, decision to publish, or preparation of the manuscript.”

**Reviewers' Comments:**

**Reviewer #1:**

The authors demonstrate the successful adaptation of the treatment of Anopheles darlingii mosquitoes with ATQ to reduce or eliminate their infection with Plasmodium vivax parasites. This is an important finding that has implications in malaria control strategies, especially in South America where this work was conducted. Moreover, it is encouraging that one of the same lead compounds identified by Paton et al (ATQ) works similarly for both of the major human-infectious malaria species.

There are no mentions of how many biological or technical replicates were conducted for each experimental type in the materials and methods, results, or figure legend. Because of this, it is unclear how rigorous this work is and how the statistical tests were run. Please include this information in a revised manuscript so that reviewers can understand and appropriately interpret the experiments and results of these statistical tests.

Answer’s author: This information has been added to the Statistic Analysis section (Lines 796 to 797), S1C Table, and the respective figure legend.

Lines 186-187: Please revise this statement as it does not agree with your findings.

Answer’s author: The sentence has been corrected (Lines 237 to 239).

Lines 226-230: Please clarify this statement, as MQ doesn’t retain partial transmission.
Answer’s author: The sentence has been corrected (Lines 297 to 301)

Clarify the group identifiers in Table 1

Answer’s author: The information has been added (Lines 381 to 385)

Italicize gene names and Latin words throughout

Answer’s author: Sorry, but we have checked, and no gene or Latin words were found without italics.

**Reviewer #2:**

This proof-of-concept paper expands the potential use of antimalarials to impair P. vivax development within one of the natural vectors, An. darlingi, by tarsal exposure, which was previous reported for P. falciparum- An. gambiae s.s. model. The validation of the use of antimalarials to blocking malaria transmission is crucial to and brings new insights into transmission dynamics in the Americas.
Experiments were robustly designed to test the TBA and TBR in several antimalarial compounds, including Atovaquone, Mefloquine, Tafenoquine and Cloroquine (some of them of extended use in Brazil as antimalarial treatment) via mosquito feeding experiments with natural P. vivax infected-blood (blood donors). One of the key findings is that Atovaquone inhibits the development of the parasite in the midgut completely after 60min exposure, and a significantly decrease after 6 min exposure to the compound (which is more realistic in mosquito behaviour).

The results obtained from these robustly designed and executed experiments supported very well their conclusion. Heterogeneity in the experiments, particularly the source of vivax gametocytes and the infectiousness between individuals, however, needs to be taken into account when evaluating the results. Furthermore, it would be good to mention the potential impact of this vector control strategy in a context as the Amazon and in *An. darlingi*, in terms of resting behaviour (very plastic and usually not indoors, so the delivery of this methodology could be challenging with certain mosquito populations) and/feeding behaviour (ATSB in the Amazon context could be challenging as potentially many other sugar sources are available for mosquitoes).

Answer’s author: Thank you for this valuable feedback and highlighting these important considerations. We have revised the manuscript to address your points as follows:

1. Regarding experimental heterogeneity, we added a statement in the Results acknowledging that natural heterogeneity between donors is an inherent feature of *P. vivax* DMFA studies and have framed our results within this context (Lines 160 to 164).
2. Regarding vector control context in Amazon, we thank the reviewer for raising this critical point about implementation. We have expanded the Discussion section (Lines 651 to 656) to specifically address the challenges for this strategy in the Amazon region.

As in some experiments the number of mosquitoes is low, it would be good to know whether there was any difference in the blood-fed females (% fed-females) between control and antimalarials experiments.
As is generally the case when using DMFA with *P. vivax*, controlling for the gametocytemia in these kinds of experiments is extremely complicated, as compared with *P. falciparum* with available cultures. It would be good whether the authors could explain whether this could be a limitation to interpret the results, particularly in prevalence and oocyst density.

Answer’s author: The feeding rate (% fed-females) in our colony is typically between 80-98%. However, during the initial contact assay experiments, the feeding rates were notably low (S1B Table). As the experiments progressed, we observed an improvement in feeding rates, and no consistent diference was observed between the control and experimental groups (S1B Table). Regarding the gametocytemia, this data is provided in S1B and C Table and is also described in the Results section (Lines 160 to 164).

Also, in some experiments with the ATQ60mins, the number of mosquitoes exposed/tested was really low (e.g. Exp1) and it would be difficult to draw any conclusions. The number of replicates by antimalarials and experiments is only in the Supplemental information. I would recommend including this information in the text or in the Figures, as it is not easy to disentangle if the figures in the graphs originated from single experiments (in general, the number of mosquitoes tested/dissected per DMFA is low). Also, for clarification purposes, it would be good to know if the same blood donor (same gametocytemia) was used across several experiments, for instance, in Fig 4. experiments in AB and CD.

## Answer’s author: This information has been added to the Statistic Analysis section (Lines 796 to 797), S1C Table, and the respective figure legend. Regarding the use of the same blood donor (with the same gametocytemia), across experiments, it was not possible to use a single donor for all DMFAs, but only for some, as is the case for the experiments in Fig 4 (S1B Table).

The authors explore the ATQ mechanism in the parasite development via *in silico* analysis. Part of this could be moved to the results sections, to make the discussion clearer.

Answer’s author: The suggested has been addressed (Lines 430 to 447).

-Please, review consistencies in Figures (font size, height graphs)
Answer’s author: The attended to the solicitation

-S2 and S3 Table: check references

Answer’s author: The references have been checked.

**Reviewer #3:**

The effect of mosquito exposure to antimalarial compounds (particularly atovaquone) on *Plasmodium* development has been recently demonstrated in *Anopheles gambiae* and *An. coluzzii* infected with *P. falciparum*. The present study represents an important contribution to the field of malariology by showing that atovaquone (and, to a lesser extent, mefloquine) similarly disrupts the development of *P. vivax* in *Anopheles darlingi*. Overall, this is a clear, well-organized, and well-written manuscript.
I have four major comments, followed by several minor remarks below.

1/ Throughout the text (e.g. line 75, 108, 128), the authors refer to the exposure/administration route as “tarsal contact". However, as in similar studies (e.g. Paton et al. 2019), the experimental setup involves placing whole mosquitoes on a treated surface, meaning that several body parts (tarsi, ventral abdomen, thorax, proboscis) may be in contact with the compound. The exclusive involvement of tarsal uptake has not been demonstrated. I guess what the author means by "tarsal contact" is kind of a standardized exposure via resting on a treated surface, not an mechanistic proof exclusive absorption through tarsi.
I suggest rephrasing to indicate that the full body of mosquitoes were exposed (by resting on treated surfaces), rather than implying that absorption occurs solely through the tarsi. Clarifying this point would avoid overstating the mechanistic interpretation of the exposure route.

**Answer’s author**: Thank you for your observation, we agreed with you. The sentences have been changed (Lines 75 to 77, 119 and 139)

2/ The authors use the term “intensity of infection” to describe the number of parasites (oocysts) per mosquito, including individuals with zero oocysts. According to standard parasitological definitions (Bush et al. 1997, J. Parasitol. 83:575–583), intensity refers specifically to the number of parasites per infected host, whereas abundance refers to the number of parasites per examined host, including uninfected individuals. In Figure 1A, since mosquitoes with zero oocysts are included (as indicated by data points at y = 0), the metric plotted corresponds to parasite abundance, not intensity.
Moreover, because abundance integrates both infected and uninfected mosquitoes, the information it conveys partly overlaps with that shown in the prevalence pie charts below (in all figures). Both parasite abundance (upper part of the pannels) and prevalence (pie chart) reflect the proportion of uninfected mosquitoes (zeros) in the dataset, meaning that they are not independent metrics. Presenting both can therefore be misleading, as the same underlying information is represented twice, once as a binary infection outcome and once as zeros within the count data.
This overlap is precisely why two-part (hurdle) models are commonly applied in parasitological analyses. These models treat infection as a two-step process:
- a binomial component (infection status: 0 = uninfected, 1 = infected), which models prevalence;
- a zero-truncated count component (e.g., negative binomial or Poisson), which models intensity among infected hosts only.
From a visual and analytical standpoint, it would be clearer to align the figures with this framework by either:
- presenting prevalence (binary outcome, analyzed with a binomial GLMM) and intensity (counts excluding zeros, analyzed with a zero-truncated negative binomial GLMM) as separate but complementary panels; or
- presenting abundance (counts including zeros, analyzed with a zero-inflated negative binomial GLMM) alone (in which case the prevalence plot becomes redundant).
Note that in the stat analyses incorporating replicate/DMFA ID and/or parasite isolate ID as random effects is required.

This distinction also carries important biological meaning: prevalence can reflects a qualitative resistance trait, the ability of the mosquito or compound to prevent parasite establishment (and/or the inability of the parasite to establish); whereas intensity reflects a quantitative resistance trait, the ability to limit parasite development once infection is established.
Adopting these definitions and ensuring consistent terminology throughout the manuscript would improve both the biological interpretation and the statistical rigor of the study.
Finally, in the same vein, please ensure that TRA is correctly estimated. TRA should be calculated based on intensity (excluding zeros), not abundance (which includes zeros), since using abundance would confound the effects of TRA with those of TBA.

Answer’s author: In response to your comment, we have clarified our statistical methods. Zero values were excluded from the analysis of oocyst intensy. In contrast, for the calculation of Transmission-Reducing Activity (TRA) – which is based on the mean oocyst density across all mosquitoes – zero values from unifected mosquitoes were included. This aligns with the standard formula used by Sagaria et al. (2023) (<https://www.sciencedirect.com/science/article/abs/pii/S1473309923002761?via%3Dihub#cesec20>), they measured the TRA as: “At least 20 mosquitoes were dissected approximately 1 week after feeding to count infected mosquitoes and parasites (oocysts) per infected mosquito. Transmission-reducing-activity was defined as ((mean-oocyst-count in control sera – mean oocyst count in test sera)/mean-oocyst-count in control sera) × 100.”

We added the information about how we calculated the TBA and TRA in the Statistics analysis section (Lines 779 to 790).

3/ Mosquito survival, Table 1. Although overall mosquito survival was relatively high (>85%), reporting only the percentage of dead mosquitoes at day 7 provides limited insight into the dynamics of survival over time. A Kaplan–Meier survival curve, showing the proportion of surviving mosquitoes each day from day 1 to day 7, would be far more informative. This approach would also allow for a proper statistical comparison between treatments using standard survival analysis methods (e.g., the Cox proportional hazards model). Presenting only day-7 mortality may mask subtle differences that occur earlier or later in the observation period.

**Answer’s author**: Thank you for your observation. Actually the analysis were done by Kplan-Meier survival curve, but the table information was incorrected. We have corrected the information and added the Cox proportional hazards model to confirm the results as supplementary data (Lines 362 to 364).

4/ Clarity on parasite isolates and experimental design. It is not clear from the Results, Figures, or Materials and Methods whether results presented in the different figures are based on the same or different *P. vivax* isolates. The correspondence between figure panels, isolate numbers, and experimental replicates is confusing. More generally, it would be helpful to specify how many isolates were used for each assay or experiment. In the Materials and Methods (lines 418–419), the authors state that “25–50 female *An. darlingi* mosquitoes, 3–5 days old, were used for each experimental and control group.” However, in e.g. Fig 1A, the sample sizes indicated are n = 80 (control) and n = 76 (ATQ_60 group). This suggests that data from several DMFA replicates or parasite isolates may have been pooled, but this is not explicitly stated. If this interpretation is correct, it would be important to clarify it in the Materials and Methods section and/or in the figure legends. Currently, it seems that one has to search through the Supplementary Tables to infer this information.
In addition, I could not find any information about the gametocytemia or parasitemia of the volunteer donors from whom the *P. vivax* isolates were obtained. These data are important, as both TBA and TRA can be influenced by the initial parasite exposure level. Reporting this information would strengthen the interpretation of the results, for example, by allowing the reader to assess whether ATQ is more effective at low versus high gametocytemia (= more effective at low vs. high infection level in the mosquito provided there is a positive relationship between gametocytemia and infection levels).

Answer’s author: Experimental replicates data have been added to the Statistic Analysis section (Lines 796 to 797), S1C Table, and the respective figure legend. Regarding the gametocytemia, this data is provided in S1B and C Table and is also described in the Results section (Lines 160 to 164). Additional, the information of Materials and Methods, the authors state that “25–50 female *An. darlingi* mosquitoes, 3–5 days old, were used for each experimental and control group.” has been corrected (Lines 683 to 684).

- line 76: "Mefloquine was also reduced parasite intensity via tarsal contact". Shouldn't this read "Mefloquine also reduced parasite intensity via tarsal contact" ?

Answer’s author: The sentence has been corrected (Line 77)

-Lines 194-197: It is unclear whether this immunflorescence assay was conducted on mosquito exposed to ATQ, pre- or post-infection?
Answer’s author: Actually, the immunflorescence assay was conducted on mosquito exposed to ATQ for 6 m pre-infection, as described in Figure 2. This information has been added to the legend (Lines 239 to 241) and the text (Lines 246 to 248).

- Line 205: "The antimalarials PQ, TQ and CQ and the compound did not achieve blocking or reduction of infection (Fig 3A, B and C)" what the term compound refer to? should this read: "The antimalarials PQ, TQ and CQ and MQ did not achieve blocking or reduction of infection (Fig 3A, B and C)". Or do you mean NGC? please clarify.

Answer’s author: Thank you for your observation. We have removed NGC because the data for this compounds is presented in another section, entitled “Tarsal exposure of *Anopheles darlingi* to Nanchangmycin (NCG) does not reduce infection with *Plasmodium vivax*”. The current section focuses exclusively on exposure to antimalarials (Lines 258 to 259)

- Line 225: "When added to the infected blood prior to infection, blocked/reduced oocysts development at the 7th day post-DMFA (Fig 4A), and sporozoite infection at the 14th day post-DMFA (Fig 4B). Shouldn't this read: "When added to the infected blood prior to infection, ATQ blocked/reduced oocysts development at the 7th day post-DMFA (Fig 4A), and sporozoite infection at the 14th day post-DMFA (Fig 4B).
Answer’s author: Thank you for your observation. The sentence has been corrected (Lines 287 to 297)

- Line 263: "Since some compounds, in addition to affecting Plasmodium development, may also impact the survival or overall fitness of mosquitoes. Therefore, we also assessed the...", shouldn't this read: "Since some compounds, in addition to affecting Plasmodium development, may also impact the survival or overall fitness of mosquitoes, we also assessed the..."
Answer’s author: The sentence has been corrected (Lines 360 to 362)

- Line 374: "Alternative delivery methods, such as sugar baits (ATSBs), could be explored such compounds" should read: "Alternative delivery methods, such as sugar baits (ATSBs), could be explored for such compound"

Answer’s author: The sentence has been corrected (Lines 614 to 615)

- Lines 375–376: The mention of nanchangmycin feels somewhat abrupt and disconnected from the surrounding text. It is not clear how this sentence fits within the current flow of the discussion. The authors should clarify the purpose of introducing nanchangmycin at this point, what specific point are they trying to make with this? If relevant, this part should be further developed and ideally presented as a separate paragraph, since, as currently written, it appears unrelated to the preceding discussion
Answer’s author: We agreed with you. We have therefore changed the order of the sentences to improve the flow and coherence of dicussion (Lines 599 to 614)

Lines 415–416: “Female *An. darlingi* mosquitoes were obtained from the colony established at the Malaria Vectors Production and Infection Platform (PIVEM), located at FIOCRUZ-RO, Brazil, as described by Araujo et al.”. Beyond the cited reference (Araujo et al. [48)), it would be useful to provide additional information about this colony: How old is it, and is it maintained as an outbred population? Furthermore, to what extent does the genetic diversity of this laboratory colony reflect that of natural An. darlingi populations? Is it sympatric with the parasite isolates? Clarifying these points would help readers assess how representative the experimental mosquitoes are of field populations and how generalizable the findings may be.

Answer’s author: Information about the *Anopheles darlingi* colony has been added as suggested (Lines 679 to 680).

-Lines 499-501: "Experiments in which the controls presented medians lower than 2.5 oocysts per mosquito and infection prevalence below 60% were not taken into consideration in the statistical analyses (S1A and S1B Table)." The rationale for this exclusion criterion is not explained. Why were experiments with lower infection levels discarded, and how is this decision justified? Excluding data based on control infection intensity or prevalence could introduce bias or affect the robustness of the analysis. Please clarify the biological or statistical reasoning behind this threshold.

Answer’s author: We thank the reviewer for this important question. We have corrected a terminology error in our manuscript (Lines 776 to 779). We incorrectly used the word “medians” when we should have used “means”.

The rationale for this exclusion criterion is twofold, based on both biological feasibility and statistical power, and is a well-established practice in mosquito feeding assay (DMFAs), particularly with *P. vivax* [50], as previously described in our work [Andrade et al. 2023, 48]. Biological justification (threshold for robust infection): a minimum level of infection in the control group is essential to confidently evaluate the transmission-blocking potential of a compound. Very low infection levels (e.g., mean < 2.5 oocysts and prevalence < 60%) are often indicative of suboptimal experimental conditions, such as low gametocyte viability in the donor blood, which can lead to highly variable and unreliable results. In such cases, a observed reduction in the treatment group could be misinteroreted as a drug effect when it might simply be a consequence of a failed or weak infection. Our chosen threshold ensures we are aanalyzing experiments where the mosquito-parasite combination was robust enough to realiably detect a true inhibitory effect. Statistical and Methodological Precedent: this approach is not arbitrary and is supported by the field. We directly adopted this specific threshold (mean < 2.5 oocysts and prevalence <60%) from the metodological framework established by Miura et al. 2020 [50]. In this kwy reference, the authours justify this exact criterion to define a “successful” *P. vivax* DMFA, stating that it ensures a sufficient infection burden to accurately measure transmission-blocking activity. By aligning our methods with this established benchmark, we aim to maintain consistency and rigor in our analyses. All experiments, including those excluded, are fully reported in S1A and S1B Tables for complete transparency. We have also rephased the sentence and added the references (Lines 776 to 779).

-Fig4A: "control" instead of "controle"

Answer’s author: The word has been corrected.
