# Supplementary material for: Anti-malarial contact dependent blocking of transmission of Plasmodium vivax by Anopheles darlingi mosquito vector
Source: PLoS Pathog. 2026 Jul 2;22(7):e1013531. doi: 10.1371/journal.ppat.1013531 (PMC13327285; doi:10.1371/journal.ppat.1013531)
Supplement: Attachment — Submitted filename: Comments from the Editor_20260310.docx [file ppat.1013531.s011.docx]

Dear Editor-in-Chief and Referees:

Thank you for your kind letter of “Decision on your submission PPATHOGENS-D-25-02262R1” on the March 5^th^, 2026. The title of the manuscript is: “**Anti-malarial contact dependent blocking of transmission of *Plasmodium vivax* by *Anopheles darlingi* mosquito vector**” by Jéssica E. A. Kassupá, Alice O. Andrade, Alessandra S. Bastos, Gabriel L. L. Moura, Marina L. Rocha, Leandro do Nascimento Martinez, Elisandra Kathellen da Silva Afonso, Wallyson Costa, Daniel Y. Bargieri, Carolina Bioni Garcia Teles, Jansen F. Medeiros, Nirbhay Kumar, Ana Carolina Ramos Guimarães, Douglas G. Paton, Flaminia Catteruccia, and Maisa S. Araujo

We are very grateful to your and the reviewers’ comments and thoughtful suggestions. We have reviewed and made careful modifications to the original manuscript and carefully proof-read the manuscript to minimize errors. We believe that the manuscript has been greatly improved and hope it has reached your journal’s standard.

Sincerely yours

Maisa S. Araújo

Here are our responses to the reviewers’ comments and list of corrections and all revisions made to manuscript was marked up using the “Track Changes” as suggested.

**Journal Requirements:**

1) We do not publish any copyright or trademark symbols that usually accompany proprietary names, eg ©,  ®, or TM  (e.g. next to drug or reagent names). Therefore please remove all instances of trademark/copyright symbols throughout the text, including:
- ® on page: 22.- ® on page: 22.

Answer’s author: We deleted it (Line 652).

**Reviewers' Comments:**

**Reviewer #1:**

The authors have considered and responded to many of the comments and requests from the reviewers, but several issues remain to be resolved so that the readers of this work can understand and confidently interpret the results.

1. It remains unclear if what is shown are biological replicates or technical replicates. They are listed as "independent replicates" or "Number of Replications" in the supplement. Please define how these replicates were done, and whether they are biological or technical replicates. If they are biological replicates, please note how many technical replicates were done per biological replicate. This is essential so that the reader knows how rigorous the work is, and what the statistical tests can or cannot say. If PLoS has standard definitions for biological and technical replicates (other journals do), please use the PLoS definitions.

Answer’s author: This information has been added to the Statistics Analysis section (Line 691), S1C Table, and the respective figure legend. Our studies were composed of up to seven independent biological replicates each consisting of evaluation of parasites stages in up to 27 mosquitoes. Note that, due to limitations imposed by high-containment and specialized environmental requirements for mosquitoes infected with human-infectious *Plasmodium* parasites, technical replicates within bioreps are typically not feasible. To ameliorate this limitations *a priori* power analyses were carried out to ensure that individual bioreps had sufficient sample size to achieve a power (1-ß) ≥0.9. We have added text to this, effect in the methods section (Lines 692 to 696)

2. The median values for Figure 4A, C, and D have changed, but the N and prevalence of infection have not. Why?

Answer’s author: Thank you for your observation. You are correct - we continued to use figures that included with zero-oocyst mosquitoes. We apologize for this mistake. We have now replaced them with the correct figures, which exclude all zero- oocyst mosquitoes.

Nonetheless, the N of infection prevalence remains unchanged, because the prevalence is calculated as the percentage of infected mosquitoes using the following formula:

% prevalence rate = 100 X (number of positive mosquitoes/ total of dissected mosquitoes).

The total number of dissected mosquitoes refers to all mosquitoes examined at 7 or 14 days post-infection (dpi), which includes both *Plasmodium* positive and negative mosquitoes (see reference 53 of the manuscript).

3. Figure 4C and 4D: the differences between control and treated samples are not even 2-fold different. While the appropriate statistical test was used (as the data was not shown to be normally distributed), and there was a statistically significant difference between groups, it is unlikely that there is a biologically meaningful difference between groups. The statements made about these differences should be revisited, as a strong conclusion was given and this compound is unlikely to be a viable intervention.

Answer’s author: It is true that the overall observed reduction in infection intensity both in terms of oocyst and sporozoites, it not as dramatic as what was observed with ATQ, we would strongly argue that this is indeed biologically significant. 1) from a clinical signifciance perspective, in our experimental system, infectious blood is resuspended in *Plasmodium* naïve serum which leads to artificially high infection intensities that can be 10- to 100- times stronger that those typically observed in the field. Thus, under “field-like”conditions, a near 2-fold reduction in parasite burden may indeed have significant negative effects on malaria transmission, even without the total elimination of the parasite from the mosquito. 2) from a bilogical significance perspective, this is the first demonstration that mefloquine has any inhibitory effect on human malaria parasites during sporogony. Given the mechanism of action of MFQ in asexual parasites is associated with disruption in heme detoxification which is not required during sporogony, our observations may point to new- or additional vulnerabilities of the parasite during mosquito development. Thus, this is a result that we feel is worthy of highlighting here, and certainly worthy of follow-up.

We acknowledge that as submitted, out position on the above was not clearly articulated, and we have expanded our discussion of the MFQ data at line 304 to 309 in the results.

4. All of the supplemental files should be reviewed and revised to better describe what is being listed so that the reader can unambiguously know what each value means. For instance, I am assuming that in Supplemental Table 1A that "M" stands for an individual mosquito that was accessed. If true, some of these experiments have a very low number of mosquitoes that were assessed (and retained in the final analyses), as noted previously by Reviewer 3. Because this experiment is weighted the same as other experiments with more mosquitoes (but still not with a large number of mosquitoes), there is an outsized effect on the final calculation by just these 4 individual mosquitoes. I'm not sure that this has been adequately addressed in the revision.

Answer’s author: Thank you for your observation. All supplemental files have been reviewed, and we enhanced the descriptions of each table and figure. The “M” means individual mosquitoes.

Regarding the number of mosquitoes per biological replicates, the only experiment that included only four mosquitoes in the analyses was Exp 1, 60 min, ID 9799, and this has now been excluded from the anlyses. Thank you for your observation.

5. I'll defer to the editor on whether the material and methods and references found on Supp Tables 3, 4, and 5 should be incorporated into the manuscript, or if they can remain in the table itself (once revised to remove mark-ups).

Answer’s author: We prefer to keep these tables as supplementary data. However, if you and editor believe they would be better incorporated into the manuscript, we are prepared to do so.

**Reviewer #3:**

1. While the authors have satisfactorily addressed two of my previous main comments (comments 1 and 4), I find that the responses to the remaining two comments (initially comments 2 and 3) remain insufficient and require further clarification and revision (see below part III minor issues).

Answer’s author: Thank you for your feedback. We apologize that our previous response to comments 2 and 3 was insufficient. We have carefully reviewed both coments again and have revised the manuscript accordingly.

2. First, the justification for including zeros in the calculation of transmission-reducing activity (TRA) is not supported by the cited reference. The quotation from Sagaria et al. (2023) explicitly states that oocysts were counted per infected mosquito. There is no indication in this definition or formula that uninfected mosquitoes (zeros) were included in the calculation of mean oocyst counts. On the contrary, the wording indicates that the metric refers to parasite intensity among infected mosquitoes only. Consequently, this reference cannot be used to justify the inclusion of zeros without redefining the biological meaning of TRA, which would then inherently conflate effects on prevalence (TBA) and effects on parasite development (TRA).

I acknowledge that some earlier studies have indeed estimated TRA using parasite abundance (i.e. including uninfected mosquitoes), a practice that can appear tempting when transmission-blocking activity (TBA) is strong, as few infected mosquitoes may remain to estimate “strict” TRA based on intensity alone. However, this methodological choice has important consequences for biological interpretation. When zeros are included, the resulting metric inherently conflates effects on parasite establishment (TBA) with effects on parasite development among infected mosquitoes (TRA), for the reasons outlined in my initial comment. If the authors choose to retain this approach, it is therefore essential that it is stated explicitly in the Methods and that readers are clearly warned that, under this definition, TRA should not be interpreted independently from TBA.
Second, the terminology used in the manuscript and figures is still problematic. The data shown include mosquitoes with zero oocysts, which corresponds to parasite abundance, not intensity as defined in standard parasitology. Even if the term “intensity” has sometimes been used loosely in the malaria literature, clear definitions exist, and adopting them would substantially improve clarity and biological interpretation. I therefore strongly recommend replacing “oocyst intensity” with “oocyst abundance” throughout the manuscript wherever zeros are included.

Answer’s author: Thank you for your observation. This discussion is important for defining the correct terminology to use when working with transmission-blocking assays. Since our group began working with TBA and TRA, we have been careful to use correct terms and calculations. We agree that citing Sagara et al. (2023) was not the most appropriate reference to demonstrate that zero oocyst counts are included in the TRA calculation. Following your comment, we contacted the senior author of the Sagara et al. (2023) paper, Dr. Patrick Duffy, to confirm that zero oocyst counts should be included in the TRA calculation. He confirmed that this correct. Specifically, TRA is calculated as 100 × {1 − (mean number of oocysts in the test group)/(mean number of oocysts in the control groups)}. This calculation includes zero oocysts counts, as described in Miura et al. (2016) (<https://www.sciencedirect.com/science/article/pii/S0264410X16305175?via%3Dihub>) and Delves et al. (2019) (<https://www.frontiersin.org/journals/microbiology/articles/10.3389/fmicb.2019.02134/full>). For further confirmation, please see the **Sup table 3 Pb feeds (Table 1)** of Delves et al. (2019), which follows the same calculation method used in our study.

You are correct that some of our figures showed zero oocyst in the infection intensity data. We have now corrected this mistake.

Additionaly, we have now provided more detailed information in **Statistics Analysis** section regarding how TBA and TRA are calculated (including zero oocyst counts), how infection intensity is calculated (excluding zero oocyst counts) and, how prevalence is calculated. These changes can be found in Lines 703 to 706 Of the revised manuscript. We hope that the revisions described above and detailed in the revised manuscript now address your concerns more clearly.

3. I appreciate the authors’ clarification and the addition of a Cox proportional hazards analysis. However, the response does not fully address my initial concern. While the authors state that Kaplan–Meier analyses were performed, the corresponding survival curves are not presented in the manuscript, and survival is still primarily summarized as percentage mortality at day 7 in Table 1.
Reporting survival at a single time point provides limited information and may mask temporal differences between treatments. If survival was analyzed using Kaplan–Meier curves and Cox models, these results should be presented in the main figures to allow readers to visualize survival dynamics over time and to ensure consistency between data presentation and statistical analyses. I therefore reiterate that Kaplan–Meier survival curves (day 1–7), together with the associated statistical comparison, should be included in the manuscript rather than relying solely on day-7 mortality values.

Answer’s author: We have added survival curves as a new Figure (Figure 6) in the manuscript, along with the corresponding statistical comparisons (long-rank test) as Table 1, no more as sup S2 Table.
