# Supplementary material for: Anti-malarial contact dependent blocking of transmission of Plasmodium vivax by Anopheles darlingi mosquito vector
Source: PLoS Pathog. 2026 Jul 2;22(7):e1013531. doi: 10.1371/journal.ppat.1013531 (PMC13327285; doi:10.1371/journal.ppat.1013531)
Supplement: Peer review history

# Peer review: Decision Letter 0

**DOI:** 10.1371/journal.ppat.1013531.r001

**Authors:** Kenneth Vernick, Jeffrey D Dvorin

4 Nov 2025

PPATHOGENS-D-25-02262

Anti-malarial contact dependent blocking of transmission of Plasmodium vivax by Anopheles darlingi mosquito vector

PLOS Pathogens

Dear Dr. Araujo,

Thank you for submitting your manuscript to PLOS Pathogens. After careful consideration, we feel that it has merit but does not fully meet PLOS Pathogens's publication criteria as it currently stands. Therefore, we invite you to submit a revised version of the manuscript that addresses the points raised during the review process.

Please submit your revised manuscript within 60 days Jan 03 2026 11:59PM. If you will need more time than this to complete your revisions, please reply to this message or contact the journal office at plospathogens@plos.org. When you're ready to submit your revision, log on to https://www.editorialmanager.com/ppathogens/ and select the 'Submissions Needing Revision' folder to locate your manuscript file.

Please include the following items when submitting your revised manuscript:

* A rebuttal letter that responds to each point raised by the editor and reviewer(s). You should upload this letter as a separate file labeled 'Response to Reviewers'. This file does not need to include responses to any formatting updates and technical items listed in the 'Journal Requirements' section below.

* A marked-up copy of your manuscript that highlights changes made to the original version. You should upload this as a separate file labeled 'Revised Manuscript with Track Changes'.

* An unmarked version of your revised paper without tracked changes. You should upload this as a separate file labeled 'Manuscript'.

If you would like to make changes to your financial disclosure, competing interests statement, or data availability statement, please make these updates within the submission form at the time of resubmission. Guidelines for resubmitting your figure files are available below the reviewer comments at the end of this letter.

We look forward to receiving your revised manuscript.

Kind regards,

Kenneth Vernick

Academic Editor

PLOS Pathogens

Jeffrey Dvorin

Section Editor

PLOS Pathogens

Sumita Bhaduri-McIntosh

Editor-in-Chief

PLOS Pathogens

orcid.org/0000-0003-2946-9497

Michael Malim

Editor-in-Chief

PLOS Pathogens

orcid.org/0000-0002-7699-2064

**Journal Requirements:**

If the reviewer comments include a recommendation to cite specific previously published works, please review and evaluate these publications to determine whether they are relevant and should be cited. There is no requirement to cite these works unless the editor has indicated otherwise.

1) We do not publish any copyright or trademark symbols that usually accompany proprietary names, eg ©,  ®, or TM  (e.g. next to drug or reagent names). Therefore please remove all instances of trademark/copyright symbols throughout the text, including:

- ® on page: 22.

2) Some material included in your submission may be copyrighted. According to PLOSu2019s copyright policy, authors who use figures or other material (e.g., graphics, clipart, maps) from another author or copyright holder must demonstrate or obtain permission to publish this material under the Creative Commons Attribution 4.0 International (CC BY 4.0) License used by PLOS journals. Please closely review the details of PLOSu2019s copyright requirements here: PLOS Licenses and Copyright. If you need to request permissions from a copyright holder, you may use PLOS's Copyright Content Permission form.

Please respond directly to this email and provide any known details concerning your material's license terms and permissions required for reuse, even if you have not yet obtained copyright permissions or are unsure of your material's copyright compatibility. Once you have responded and addressed all other outstanding technical requirements, you may resubmit your manuscript within Editorial Manager.

Potential Copyright Issues:

i) Figures 1A, 1C, 2A, 2B, 3, 4, and 5. Please confirm whether you drew the images / clip-art within the figure panels by hand. If you did not draw the images, please provide (a) a link to the source of the images or icons and their license / terms of use; or (b) written permission from the copyright holder to publish the images or icons under our CC BY 4.0 license. Alternatively, you may replace the images with open source alternatives. See these open source resources you may use to replace images / clip-art:

- https://commons.wikimedia.org

- https://openclipart.org/.

3) Please amend your detailed Financial Disclosure statement. This is published with the article. It must therefore be completed in full sentences and contain the exact wording you wish to be published.

1) State the initials, alongside each funding source, of each author to receive each grant. For example: "This work was supported by the National Institutes of Health (####### to AM; ###### to CJ) and the National Science Foundation (###### to AM)."

2) State what role the funders took in the study. If the funders had no role in your study, please state: "The funders had no role in study design, data collection and analysis, decision to publish, or preparation of the manuscript."

3) If any authors received a salary from any of your funders, please state which authors and which funders..

If you did not receive any funding for this study, please simply state: u201cThe authors received no specific funding for this work.u201d

4)  Please ensure that the funders and grant numbers match between the Financial Disclosure field and the Funding Information tab in your submission form. Note that the funders must be provided in the same order in both places as well.

**Reviewers' Comments:**

Reviewer's Responses to Questions

**Part I - Summary**

Please use this section to discuss strengths/weaknesses of study, novelty/significance, general execution and scholarship.

Reviewer #1: The authors demonstrate the successful adaptation of the treatment of Anopheles darlingii mosquitoes with ATQ to reduce or eliminate their infection with Plasmodium vivax parasites. This is an important finding that has implications in malaria control strategies, especially in South America where this work was conducted. Moreover, it is encouraging that one of the same lead compounds identified by Paton et al (ATQ) works similarly for both of the major human-infectious malaria species.

Reviewer #2: This proof-of-concept paper expands the potential use of antimalarials to impair P. vivax development within one of the natural vectors, An. darlingi, by tarsal exposure, which was previous reported for P. falciparum- An. gambiae s.s. model. The validation of the use of antimalarials to blocking malaria transmission is crucial to and brings new insights into transmission dynamics in the Americas.

Experiments were robustly designed to test the TBA and TBR in several antimalarial compounds, including Atovaquone, Mefloquine, Tafenoquine and Cloroquine (some of them of extended use in Brazil as antimalarial treatment) via mosquito feeding experiments with natural P. vivax infected-blood (blood donors). One of the key findings is that Atovaquone inhibits the development of the parasite in the midgut completely after 60min exposure, and a significantly decrease after 6 min exposure to the compound (which is more realistic in mosquito behaviour).

The results obtained from these robustly designed and executed experiments supported very well their conclusion. Heterogeneity in the experiments, particularly the source of vivax gametocytes and the infectiousness between individuals, however, needs to be taken into account when evaluating the results. Furthermore, it would be good to mention the potential impact of this vector control strategy in a context as the Amazon and in An. darlingi, in terms of resting behaviour (very plastic and usually not indoors, so the delivery of this methodology could be challenging with certain mosquito populations) and/feeding behaviour (ATSB in the Amazon context could be challenging as potentially many other sugar sources are available for mosquitoes).

Reviewer #3: The effect of mosquito exposure to antimalarial compounds (particularly atovaquone) on Plasmodium development has been recently demonstrated in Anopheles gambiae and An. coluzzii infected with P. falciparum. The present study represents an important contribution to the field of malariology by showing that atovaquone (and, to a lesser extent, mefloquine) similarly disrupts the development of P. vivax in Anopheles darlingi. Overall, this is a clear, well-organized, and well-written manuscript.

I have four major comments, followed by several minor remarks below.

**********

**Part II – Major Issues: Key Experiments Required for Acceptance**

Please use this section to detail the key new experiments or modifications of existing experiments that should be absolutely required to validate study conclusions.

Generally, there should be no more than 3 such required experiments or major modifications for a "Major Revision" recommendation. If more than 3 experiments are necessary to validate the study conclusions, then you are encouraged to recommend "Reject".

Reviewer #1: There are no mentions of how many biological or technical replicates were conducted for each experimental type in the materials and methods, results, or figure legend. Because of this, it is unclear how rigorous this work is and how the statistical tests were run. Please include this information in a revised manuscript so that reviewers can understand and appropriately interpret the experiments and results of these statistical tests.

Reviewer #2: As in some experiments the number of mosquitoes is low, it would be good to know whether there was any difference in the blood-fed females (% fed-females) between control and antimalarials experiments.

As is generally the case when using DMFA with P. vivax, controlling for the gametocytemia in these kinds of experiments is extremely complicated, as compared with P. falciparum with available cultures. It would be good whether the authors could explain whether this could be a limitation to interpret the results, particularly in prevalence and oocyst density.

Also, in some experiments with the ATQ60mins, the number of mosquitoes exposed/tested was really low (e.g. Exp1) and it would be difficult to draw any conclusions. The number of replicates by antimalarials and experiments is only in the Supplemental information. I would recommend including this information in the text or in the Figures, as it is not easy to disentangle if the figures in the graphs originated from single experiments (in general, the number of mosquitoes tested/dissected per DMFA is low). Also, for clarification purposes, it would be good to know if the same blood donor (same gametocytemia) was used across several experiments, for instance, in Fig 4. experiments in AB and CD.

The authors explore the ATQ mechanism in the parasite development via in silico analysis. Part of this could be moved to the results sections, to make the discussion clearer.

Reviewer #3: 1/ Throughout the text (e.g. line 75, 108, 128), the authors refer to the exposure/administration route as “tarsal contact". However, as in similar studies (e.g. Paton et al. 2019), the experimental setup involves placing whole mosquitoes on a treated surface, meaning that several body parts (tarsi, ventral abdomen, thorax, proboscis) may be in contact with the compound. The exclusive involvement of tarsal uptake has not been demonstrated. I guess what the author means by "tarsal contact" is kind of a standardized exposure via resting on a treated surface, not an mechanistic proof exclusive absorption through tarsi.

I suggest rephrasing to indicate that the full body of mosquitoes were exposed (by resting on treated surfaces), rather than implying that absorption occurs solely through the tarsi. Clarifying this point would avoid overstating the mechanistic interpretation of the exposure route.

2/ The authors use the term “intensity of infection” to describe the number of parasites (oocysts) per mosquito, including individuals with zero oocysts. According to standard parasitological definitions (Bush et al. 1997, J. Parasitol. 83:575–583), intensity refers specifically to the number of parasites per infected host, whereas abundance refers to the number of parasites per examined host, including uninfected individuals. In Figure 1A, since mosquitoes with zero oocysts are included (as indicated by data points at y = 0), the metric plotted corresponds to parasite abundance, not intensity.

Moreover, because abundance integrates both infected and uninfected mosquitoes, the information it conveys partly overlaps with that shown in the prevalence pie charts below (in all figures). Both parasite abundance (upper part of the pannels) and prevalence (pie chart) reflect the proportion of uninfected mosquitoes (zeros) in the dataset, meaning that they are not independent metrics. Presenting both can therefore be misleading, as the same underlying information is represented twice, once as a binary infection outcome and once as zeros within the count data.

This overlap is precisely why two-part (hurdle) models are commonly applied in parasitological analyses. These models treat infection as a two-step process:

- a binomial component (infection status: 0 = uninfected, 1 = infected), which models prevalence;

- a zero-truncated count component (e.g., negative binomial or Poisson), which models intensity among infected hosts only.

From a visual and analytical standpoint, it would be clearer to align the figures with this framework by either:

- presenting prevalence (binary outcome, analyzed with a binomial GLMM) and intensity (counts excluding zeros, analyzed with a zero-truncated negative binomial GLMM) as separate but complementary panels; or

- presenting abundance (counts including zeros, analyzed with a zero-inflated negative binomial GLMM) alone (in which case the prevalence plot becomes redundant).

Note that in the stat analyses incorporating replicate/DMFA ID and/or parasite isolate ID as random effects is required.

This distinction also carries important biological meaning: prevalence can reflects a qualitative resistance trait, the ability of the mosquito or compound to prevent parasite establishment (and/or the inability of the parasite to establish); whereas intensity reflects a quantitative resistance trait, the ability to limit parasite development once infection is established.

Adopting these definitions and ensuring consistent terminology throughout the manuscript would improve both the biological interpretation and the statistical rigor of the study.

Finally, in the same vein, please ensure that TRA is correctly estimated. TRA should be calculated based on intensity (excluding zeros), not abundance (which includes zeros), since using abundance would confound the effects of TRA with those of TBA.

3/ Mosquito survival, Table 1. Although overall mosquito survival was relatively high (>85%), reporting only the percentage of dead mosquitoes at day 7 provides limited insight into the dynamics of survival over time. A Kaplan–Meier survival curve, showing the proportion of surviving mosquitoes each day from day 1 to day 7, would be far more informative. This approach would also allow for a proper statistical comparison between treatments using standard survival analysis methods (e.g., the Cox proportional hazards model). Presenting only day-7 mortality may mask subtle differences that occur earlier or later in the observation period.

4/ Clarity on parasite isolates and experimental design. It is not clear from the Results, Figures, or Materials and Methods whether results presented in the different figures are based on the same or different P. vivax isolates. The correspondence between figure panels, isolate numbers, and experimental replicates is confusing. More generally, it would be helpful to specify how many isolates were used for each assay or experiment. In the Materials and Methods (lines 418–419), the authors state that “25–50 female An. darlingi mosquitoes, 3–5 days old, were used for each experimental and control group.” However, in e.g. Fig 1A, the sample sizes indicated are n = 80 (control) and n = 76 (ATQ_60 group). This suggests that data from several DMFA replicates or parasite isolates may have been pooled, but this is not explicitly stated. If this interpretation is correct, it would be important to clarify it in the Materials and Methods section and/or in the figure legends. Currently, it seems that one has to search through the Supplementary Tables to infer this information.

In addition, I could not find any information about the gametocytemia or parasitemia of the volunteer donors from whom the P. vivax isolates were obtained. These data are important, as both TBA and TRA can be influenced by the initial parasite exposure level. Reporting this information would strengthen the interpretation of the results, for example, by allowing the reader to assess whether ATQ is more effective at low versus high gametocytemia (= more effective at low vs. high infection level in the mosquito provided there is a positive relationship between gametocytemia and infection levels).

**********

**Part III – Minor Issues: Editorial and Data Presentation Modifications**

Please use this section for editorial suggestions as well as relatively minor modifications of existing data that would enhance clarity.

Reviewer #1: Lines 186-187: Please revise this statement as it does not agree with your findings.

Lines 226-230: Please clarify this statement, as MQ doesn’t retain partial transmission.

Clarify the group identifiers in Table 1

Italicize gene names and Latin words throughout

Reviewer #2: -Please, review consistencies in Figures (font size, height graphs)

-S2 and S3 Table: check references

Reviewer #3: - line 76: "Mefloquine was also reduced parasite intensity via tarsal contact". Shouldn't this read "Mefloquine also reduced parasite intensity via tarsal contact" ?

-Lines 194-197: It is unclear whether this immunflorescence assay was conducted on mosquito exposed to ATQ, pre- or post-infection?

- Line 205: "The antimalarials PQ, TQ and CQ and the compound did not achieve blocking or reduction of infection (Fig 3A, B and C)" what the term compound refer to? should this read: "The antimalarials PQ, TQ and CQ and MQ did not achieve blocking or reduction of infection (Fig 3A, B and C)". Or do you mean NGC? please clarify.

- Line 225: "When added to the infected blood prior to infection, blocked/reduced oocysts development at the 7th day post-DMFA (Fig 4A), and sporozoite infection at the 14th day post-DMFA (Fig 4B). Shouldn't this read: "When added to the infected blood prior to infection, ATQ blocked/reduced oocysts development at the 7th day post-DMFA (Fig 4A), and sporozoite infection at the 14th day post-DMFA (Fig 4B).

- Line 263: "Since some compounds, in addition to affecting Plasmodium development, may also impact the survival or overall fitness of mosquitoes. Therefore, we also assessed the...", shouldn't this read: "Since some compounds, in addition to affecting Plasmodium development, may also impact the survival or overall fitness of mosquitoes, we also assessed the..."

- Line 374: "Alternative delivery methods, such as sugar baits (ATSBs), could be explored such compounds" should read: "Alternative delivery methods, such as sugar baits (ATSBs), could be explored for such compound"

- Lines 375–376: The mention of nanchangmycin feels somewhat abrupt and disconnected from the surrounding text. It is not clear how this sentence fits within the current flow of the discussion. The authors should clarify the purpose of introducing nanchangmycin at this point, what specific point are they trying to make with this? If relevant, this part should be further developed and ideally presented as a separate paragraph, since, as currently written, it appears unrelated to the preceding discussion

Lines 415–416: “Female An. darlingi mosquitoes were obtained from the colony established at the Malaria Vectors Production and Infection Platform (PIVEM), located at FIOCRUZ-RO, Brazil, as described by Araujo et al.”. Beyond the cited reference (Araujo et al. [48)), it would be useful to provide additional information about this colony: How old is it, and is it maintained as an outbred population? Furthermore, to what extent does the genetic diversity of this laboratory colony reflect that of natural An. darlingi populations? Is it sympatric with the parasite isolates? Clarifying these points would help readers assess how representative the experimental mosquitoes are of field populations and how generalizable the findings may be.

-Lines 499-501: "Experiments in which the controls presented medians lower than 2.5 oocysts per mosquito and infection prevalence below 60% were not taken into consideration in the statistical analyses (S1A and S1B Table)." The rationale for this exclusion criterion is not explained. Why were experiments with lower infection levels discarded, and how is this decision justified? Excluding data based on control infection intensity or prevalence could introduce bias or affect the robustness of the analysis. Please clarify the biological or statistical reasoning behind this threshold.

-Fig4A: "control" instead of "controle"

**********

PLOS authors have the option to publish the peer review history of their article (what does this mean?). If published, this will include your full peer review and any attached files.

If you choose “no”, your identity will remain anonymous but your review may still be made public.

**Do you want your identity to be public for this peer review?**  For information about this choice, including consent withdrawal, please see our Privacy Policy.

Reviewer #1: No

Reviewer #2: No

Reviewer #3: No

**Figure resubmission:**

While revising your submission, we strongly recommend that you use PLOS’s NAAS tool (https://ngplosjournals.pagemajik.ai/artanalysis) to test your figure files. NAAS can convert your figure files to the TIFF file type and meet basic requirements (such as print size, resolution), or provide you with a report on issues that do not meet our requirements and that NAAS cannot fix.

After uploading your figures to PLOS’s NAAS tool - https://ngplosjournals.pagemajik.ai/artanalysis, NAAS will process the files provided and display the results in the "Uploaded Files" section of the page as the processing is complete. If the uploaded figures meet our requirements (or NAAS is able to fix the files to meet our requirements), the figure will be marked as "fixed" above. If NAAS is unable to fix the files, a red "failed" label will appear above. When NAAS has confirmed that the figure files meet our requirements, please download the file via the download option, and include these NAAS processed figure files when submitting your revised manuscript.

**Reproducibility:**

To enhance the reproducibility of your results, we recommend that authors of applicable studies deposit laboratory protocols in protocols.io, where a protocol can be assigned its own identifier (DOI) such that it can be cited independently in the future. Additionally, PLOS ONE offers an option to publish peer-reviewed clinical study protocols. Read more information on sharing protocols at https://plos.org/protocols?utm_medium=editorial-email&utm_source=authorletters&utm_campaign=protocols

# Author comment: Author response to Decision Letter 1

**DOI:** 10.1371/journal.ppat.1013531.r002

26 Dec 2025

Attachment Submitted filename: Comments from the Editor.docx

# Peer review: Decision Letter 1

**DOI:** 10.1371/journal.ppat.1013531.r003

**Authors:** Kenneth Vernick, Jeffrey D Dvorin

5 Mar 2026

PPATHOGENS-D-25-02262R1

Anti-malarial contact dependent blocking of transmission of Plasmodium vivax by Anopheles darlingi mosquito vector

PLOS Pathogens

Dear Dr. Araujo,

Thank you for submitting your manuscript to PLOS Pathogens. After careful consideration, we feel that it has merit but does not fully meet PLOS Pathogens's publication criteria as it currently stands. Therefore, we invite you to submit a revised version of the manuscript that addresses the points raised during the review process.

Please submit your revised manuscript by May 04 2026 11:59PM. If you will need more time than this to complete your revisions, please reply to this message or contact the journal office at plospathogens@plos.org. When you're ready to submit your revision, log on to https://www.editorialmanager.com/ppathogens/ and select the 'Submissions Needing Revision' folder to locate your manuscript file.

Please include the following items when submitting your revised manuscript:

* A letter that responds to each point raised by the editor and reviewer(s). You should upload this letter as a separate file labeled 'Response to Reviewers'. This file does not need to include responses to any formatting updates and technical items listed in the 'Journal Requirements' section below.

* A marked-up copy of your manuscript that highlights changes made to the original version. You should upload this as a separate file labeled 'Revised Manuscript with Track Changes'.

* An unmarked version of your revised paper without tracked changes. You should upload this as a separate file labeled 'Manuscript'.

If you would like to make changes to your financial disclosure, competing interests statement, or data availability statement, please make these updates within the submission form at the time of resubmission. Guidelines for resubmitting your figure files are available below the reviewer comments at the end of this letter.

We look forward to receiving your revised manuscript.

Kind regards,

Kenneth Vernick

Academic Editor

PLOS Pathogens

Jeffrey Dvorin

Section Editor

PLOS Pathogens

Sumita Bhaduri-McIntosh

Editor-in-Chief

PLOS Pathogens

orcid.org/0000-0003-2946-9497

Michael Malim

Editor-in-Chief

PLOS Pathogens

orcid.org/0000-0002-7699-2064

**Additional Editor Comments :**

The two reviewers still have several issues, which can be addressed without need for new experimental work. Please address all of the points raised, but the major ones are: Rev1, clarification between whether you present biological replicates or technical replicates; and Rev3, present infection metrics that do not conflate effects on prevalence (TBA) and effects on parasite development (TRA), and improve the presentation of mosquito survival data.

**Journal Requirements:**

1) We do not publish any copyright or trademark symbols that usually accompany proprietary names, eg ©,  ®, or TM  (e.g. next to drug or reagent names). Therefore please remove all instances of trademark/copyright symbols throughout the text, including:

- ® on page: 22.

Note: If the reviewer comments include a recommendation to cite specific previously published works, please review and evaluate these publications to determine whether they are relevant and should be cited. There is no requirement to cite these works unless the editor has indicated otherwise.

**Reviewers' Comments:**

Reviewer's Responses to Questions

**Part I - Summary**

Please use this section to discuss strengths/weaknesses of study, novelty/significance, general execution and scholarship.

Reviewer #1: The authors have considered and responded to many of the comments and requests from the reviewers, but several issues remain to be resolved so that the readers of this work can understand and confidently interpret the results.

Reviewer #3: While the authors have satisfactorily addressed two of my previous main comments (comments 1 and 4), I find that the responses to the remaining two comments (initially comments 2 and 3) remain insufficient and require further clarification and revision (see below part III minor issues)

**********

**Part II – Major Issues: Key Experiments Required for Acceptance**

Please use this section to detail the key new experiments or modifications of existing experiments that should be absolutely required to validate study conclusions.

Generally, there should be no more than 3 such required experiments or major modifications for a "Major Revision" recommendation. If more than 3 experiments are necessary to validate the study conclusions, then you are encouraged to recommend "Reject".

Reviewer #1: 1. It remains unclear if what is shown are biological replicates or technical replicates. They are listed as "independent replicates" or "Number of Replications" in the supplement. Please define how these replicates were done, and whether they are biological or technical replicates. If they are biological replicates, please note how many technical replicates were done per biological replicate. This is essential so that the reader knows how rigorous the work is, and what the statistical tests can or cannot say. If PLoS has standard definitions for biological and technical replicates (other journals do), please use the PLoS definitions.

2. The median values for Figure 4A, C, and D have changed, but the N and prevalence of infection have not. Why?

3. Figure 4C and 4D: the differences between control and treated samples are not even 2-fold different. While the appropriate statistical test was used (as the data was not shown to be normally distributed), and there was a statistically significant difference between groups, it is unlikely that there is a biologically meaningful difference between groups. The statements made about these differences should be revisited, as a strong conclusion was given and this compound is unlikely to be a viable intervention.

4. All of the supplemental files should be reviewed and revised to better describe what is being listed so that the reader can unambiguously know what each value means. For instance, I am assuming that in Supplemental Table 1A that "M" stands for an individual mosquito that was accessed. If true, some of these experiments have a very low number of mosquitoes that were assessed (and retained in the final analyses), as noted previously by Reviewer 3. Because this experiment is weighted the same as other experiments with more mosquitoes (but still not with a large number of mosquitoes), there is an outsized effect on the final calculation by just these 4 individual mosquitoes. I'm not sure that this has been adequately addressed in the revision.

Reviewer #3: (No Response)

**********

**Part III – Minor Issues: Editorial and Data Presentation Modifications**

Please use this section for editorial suggestions as well as relatively minor modifications of existing data that would enhance clarity.

Reviewer #1: I'll defer to the editor on whether the material and methods and references found on Supp Tables 3, 4, and 5 should be incorporated into the manuscript, or if they can remain in the table itself (once revised to remove mark-ups).

Reviewer #3: Previous comment 2/

First, the justification for including zeros in the calculation of transmission-reducing activity (TRA) is not supported by the cited reference. The quotation from Sagaria et al. (2023) explicitly states that oocysts were counted per infected mosquito. There is no indication in this definition or formula that uninfected mosquitoes (zeros) were included in the calculation of mean oocyst counts. On the contrary, the wording indicates that the metric refers to parasite intensity among infected mosquitoes only. Consequently, this reference cannot be used to justify the inclusion of zeros without redefining the biological meaning of TRA, which would then inherently conflate effects on prevalence (TBA) and effects on parasite development (TRA).

I acknowledge that some earlier studies have indeed estimated TRA using parasite abundance (i.e. including uninfected mosquitoes), a practice that can appear tempting when transmission-blocking activity (TBA) is strong, as few infected mosquitoes may remain to estimate “strict” TRA based on intensity alone. However, this methodological choice has important consequences for biological interpretation. When zeros are included, the resulting metric inherently conflates effects on parasite establishment (TBA) with effects on parasite development among infected mosquitoes (TRA), for the reasons outlined in my initial comment. If the authors choose to retain this approach, it is therefore essential that it is stated explicitly in the Methods and that readers are clearly warned that, under this definition, TRA should not be interpreted independently from TBA.

Second, the terminology used in the manuscript and figures is still problematic. The data shown include mosquitoes with zero oocysts, which corresponds to parasite abundance, not intensity as defined in standard parasitology. Even if the term “intensity” has sometimes been used loosely in the malaria literature, clear definitions exist, and adopting them would substantially improve clarity and biological interpretation. I therefore strongly recommend replacing “oocyst intensity” with “oocyst abundance” throughout the manuscript wherever zeros are included.

Previous comment 3/

I appreciate the authors’ clarification and the addition of a Cox proportional hazards analysis. However, the response does not fully address my initial concern. While the authors state that Kaplan–Meier analyses were performed, the corresponding survival curves are not presented in the manuscript, and survival is still primarily summarized as percentage mortality at day 7 in Table 1.

Reporting survival at a single time point provides limited information and may mask temporal differences between treatments. If survival was analyzed using Kaplan–Meier curves and Cox models, these results should be presented in the main figures to allow readers to visualize survival dynamics over time and to ensure consistency between data presentation and statistical analyses. I therefore reiterate that Kaplan–Meier survival curves (day 1–7), together with the associated statistical comparison, should be included in the manuscript rather than relying solely on day-7 mortality values.

**********

PLOS authors have the option to publish the peer review history of their article (what does this mean?). If published, this will include your full peer review and any attached files.

If you choose “no”, your identity will remain anonymous but your review may still be made public.

**Do you want your identity to be public for this peer review?**  For information about this choice, including consent withdrawal, please see our Privacy Policy.

Reviewer #1: No

Reviewer #3: No

[NOTE: If reviewer comments were submitted as an attachment file, they will be attached to this email and accessible via the submission site. Please log into your account, locate the manuscript record, and check for the action link "View Attachments". If this link does not appear, there are no attachment files.]

**Figure resubmission:**

While revising your submission, we strongly recommend that you use PLOS’s NAAS tool (https://ngplosjournals.pagemajik.ai/artanalysis) to test your figure files. NAAS can convert your figure files to the TIFF file type and meet basic requirements (such as print size, resolution), or provide you with a report on issues that do not meet our requirements and that NAAS cannot fix.

After uploading your figures to PLOS’s NAAS tool - https://ngplosjournals.pagemajik.ai/artanalysis, NAAS will process the files provided and display the results in the "Uploaded Files" section of the page as the processing is complete. If the uploaded figures meet our requirements (or NAAS is able to fix the files to meet our requirements), the figure will be marked as "fixed" above. If NAAS is unable to fix the files, a red "failed" label will appear above. When NAAS has confirmed that the figure files meet our requirements, please download the file via the download option, and include these NAAS processed figure files when submitting your revised manuscript.

**Reproducibility:**

To enhance the reproducibility of your results, we recommend that authors of applicable studies deposit laboratory protocols in protocols.io, where a protocol can be assigned its own identifier (DOI) such that it can be cited independently in the future. Additionally, PLOS ONE offers an option to publish peer-reviewed clinical study protocols. Read more information on sharing protocols at https://plos.org/protocols?utm_medium=editorial-email&utm_source=authorletters&utm_campaign=protocols

# Author comment: Author response to Decision Letter 2

**DOI:** 10.1371/journal.ppat.1013531.r004

13 May 2026

Attachment Submitted filename: Comments from the Editor_20260310.docx

# Editor report: Decision Letter 2

**DOI:** 10.1371/journal.ppat.1013531.r005

**Authors:** Kenneth Vernick, Jeffrey D Dvorin

19 May 2026

Dear Dr Araujo,

We are pleased to inform you that your manuscript 'Anti-malarial contact dependent blocking of transmission of Plasmodium vivax by Anopheles darlingi mosquito vector' has been provisionally accepted for publication in PLOS Pathogens.

Before your manuscript can be formally accepted you will need to complete some formatting changes, which you will receive in a follow up email. A member of our team will be in touch with a set of requests.

Please note that your manuscript will not be scheduled for publication until you have made the required changes, so a swift response is appreciated.

IMPORTANT: The editorial review process is now complete. PLOS will only permit corrections to spelling, formatting or significant scientific errors from this point onwards. Requests for major changes, or any which affect the scientific understanding of your work, will cause delays to the publication date of your manuscript.

Should you, your institution's press office or the journal office choose to press release your paper, you will automatically be opted out of early publication. We ask that you notify us now if you or your institution is planning to press release the article. All press must be co-ordinated with PLOS.

Thank you again for supporting Open Access publishing; we are looking forward to publishing your work in PLOS Pathogens.

Best regards,

Kenneth Vernick

Academic Editor

PLOS Pathogens

Jeffrey Dvorin

Section Editor

PLOS Pathogens

Sumita Bhaduri-McIntosh

Editor-in-Chief

PLOS Pathogens

orcid.org/0000-0003-2946-9497

Michael Malim

Editor-in-Chief

PLOS Pathogens

orcid.org/0000-0002-7699-2064

***********************************************************

Reviewer Comments (if any, and for reference):

# Editor report: Acceptance letter

**DOI:** 10.1371/journal.ppat.1013531.r006

**Authors:** Kenneth Vernick, Jeffrey D Dvorin

Dear Dr Araujo,

We are delighted to inform you that your manuscript, "Anti-malarial contact dependent blocking of transmission of Plasmodium vivax by Anopheles darlingi mosquito vector," has been formally accepted for publication in PLOS Pathogens.

We have now passed your article onto the PLOS Production Department who will complete the rest of the pre-publication process. All authors will receive a confirmation email upon publication.

The corresponding author will soon be receiving a typeset proof for review, to ensure errors have not been introduced during production. Please review the PDF proof of your manuscript carefully, as this is the last chance to correct any scientific or type-setting errors. Please note that major changes, or those which affect the scientific understanding of the work, will likely cause delays to the publication date of your manuscript. Note: Proofs for Front Matter articles (Pearls, Reviews, Opinions, etc...) are generated on a different schedule and may not be made available as quickly.

Soon after your final files are uploaded, the early version of your manuscript, if you opted to have an early version of your article, will be published online. The date of the early version will be your article's publication date. The final article will be published to the same URL, and all versions of the paper will be accessible to readers.

For Research Articles, you will receive an invoice from PLOS for your publication fee after your manuscript has reached the completed accept phase. If you receive an email requesting payment before acceptance or for any other service, this may be a phishing scheme. Learn how to identify phishing emails and protect your accounts at https://explore.plos.org/phishing.

Thank you again for supporting open-access publishing; we are looking forward to publishing your work in PLOS Pathogens.

Best regards,

Sumita Bhaduri-McIntosh

Editor-in-Chief

PLOS Pathogens

orcid.org/0000-0003-2946-9497

Michael Malim

Editor-in-Chief

PLOS Pathogens

orcid.org/0000-0002-7699-2064
